# DeepQR: single-molecule QR codes for optical gene-expression analysis

**DOI:** 10.1515/nanoph-2024-0236

**Published:** 2024-07-30

**Authors:** Jonathan Jeffet, Barak Hadad, Sahar Froim, Kawsar Kaboub, Keren M. Rabinowitz, Jasline Deek, Sapir Margalit, Iris Dotan, Alon Bahabad, Yuval Ebenstein

**Affiliations:** School of Physics and Astronomy, The Raymond and Beverly Sackler Faculty of Exact Sciences, 26745 Tel Aviv University, Tel Aviv 6997801, Israel; School of Chemistry, The Raymond and Beverly Sackler Faculty of Exact Sciences, 26745 Tel Aviv University, Tel Aviv 6997801, Israel; Department of Biomedical Engineering, Fleischman Faculty of Engineering, 26745 Tel Aviv University, Tel Aviv 6997801, Israel; Center for Light Matter Interaction, 26745 Tel Aviv University, Tel Aviv 6997801, Israel; Department of Physical Electronics, School of Electrical Engineering, Fleischman Faculty of Engineering, Tel-Aviv University, Tel Aviv 6997801, Israel; Division of Gastroenterology, Rabin Medical Center, Petah Tikva, Israel; Felsenstein Medical Research Center, Faculty of Medical & Health Sciences, 26745 Tel Aviv University, Tel Aviv, Israel

**Keywords:** spectral imaging, machine learning, gene expression, RNA, single-molecule, NanoString

## Abstract

Optical imaging and single-molecule imaging, in particular, utilize fluorescent tags in order to differentiate observed species by color. The degree of color multiplexing is dependent on the available spectral detection window and the ability to distinguish between fluorophores of different colors within this window. Consequently, most single-molecule imaging techniques rely on two to four colors for multiplexing. DeepQR combines compact spectral imaging with deep learning to enable 4 color acquisition with only 3 spectral detection windows. It allows rapid high-throughput acquisition and decoding of hundreds of unique single-molecule color combinations applied here to tag native RNA targets. We validate our method with clinical samples analyzed with the NanoString gene-expression inflammation panel side by side with the commercially available NanoString nCounter system. We demonstrate high concordance with “gold-standard” filter-based imaging and over a four-fold decrease in acquisition time by applying a single snapshot to record four-color barcodes. The new approach paves the path for extreme single-molecule multiplexing.

## 1 Introduction

Gene expression analysis is a powerful tool for exploring physiological responses to environmental exposures, external stimuli, and various disease states [1]. RNA sequencing is able to characterize the full RNA content of a sample but requires reverse transcription and PCR amplification, which introduce bias to quantitative expression analysis [2]. Native RNA nanopore sequencing has recently become available and provides single-molecule information at single-base resolution. However, the method requires large amounts of input material and still suffers from technical drawbacks and high costs [3]. An outstanding goal in single-molecule analysis is to capture extensive transcriptome [4] or proteome [5] panels from small amounts of a native unamplified sample. Fluorescence detection from individual target molecules presents the ultimate sensitivity but suffers from the low multiplexing capabilities offered by standard optics. One way to increase the number of uniquely detected tags is to arrange them as a sequence of colors in a linear arrangement as introduced by NanoString Technologies, Inc. [6]. QR codes extend the information content of linear barcodes by utilizing a second dimension for encoding data. We adapt a similar concept in order to encode color information in the second dimension. We introduce DeepQR, an optical method that generates hundreds of unique molecular identifiers for RNA targets, using spectral imaging combined with machine learning-based image registration. DeepQR exploits the visible spectrum more efficiently than conventional filter-based microscopy, allowing for enhanced color multiplexing. Introducing minute spectral shifts to the detected optical point spread function (PSF) allows distinguishing between spectrally similar fluorophores in the same color channel of the microscope by uniquely shifting each color from the optical axis. Thus, a conventional scientific monochrome camera can simultaneously record multiple colors in the visible spectrum with a single snapshot.

**Figure 1:**
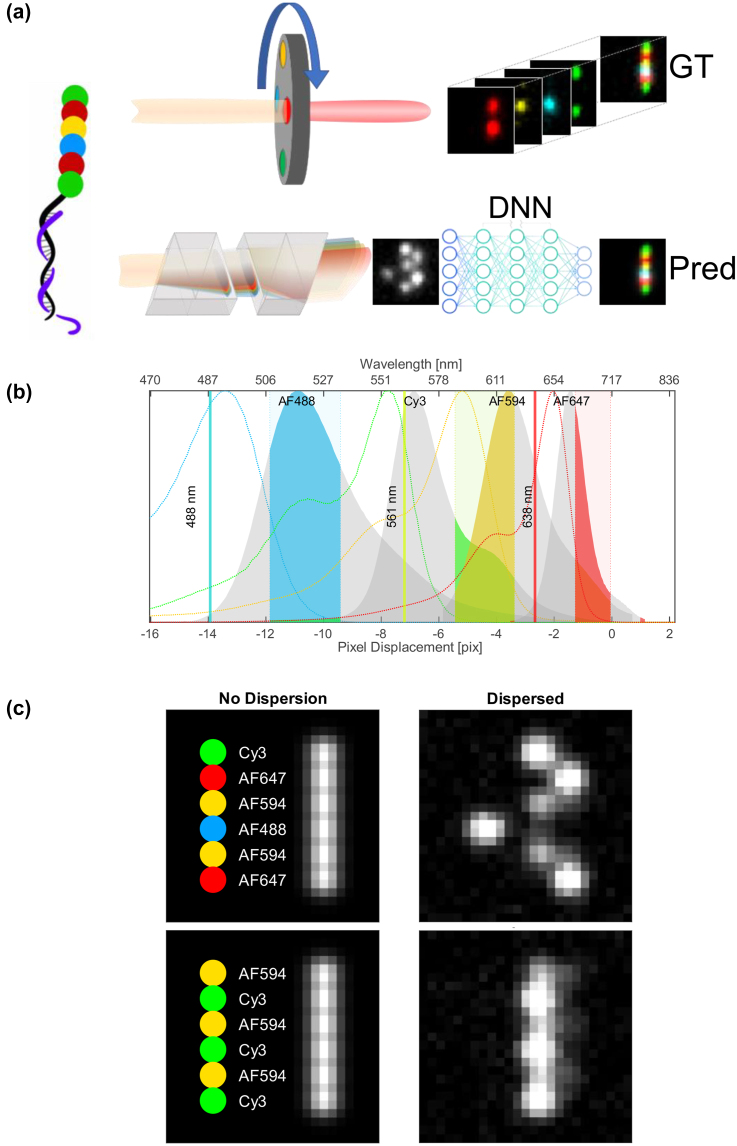
Resolving multicolor barcodes. (a) Illustration of a linear four-color barcode detected with two acquisition pipelines. Top, Standard color imaging overlaying four images acquired with dedicated emission filters to resolve the ground truth (GT). Bottom, Color image reconstruction with DeepQR: a single spectral image acquired by dispersing fluorescence emission with two direct-vision prisms is converted to a multi-color prediction image (Pred) by a deep neural network (DNN) U-Net. (b) NanoString’s fluorophores spectra overlayed with the CoCoS system’s single three-band emission filter. The three excitation lasers used for four-color detection are displayed as solid lines. Colored patches indicate the multiband emission filter channels, showing that DeepQR allows resolving the overlapping spectra of Cy3 and AF594 using a single spectral channel. (c) Simulation of two barcodes dispersed to create a spectral QR barcode. Left: The nondispersed labeled barcode, fluorophore names and colors are displayed to the left of the simulated PSF. Right: The dispersed barcode allowing to interpret its composition with its unique spectral PSF. Using four fluorophores per barcode allows to tag up to 972 unique RNA targets.

To demonstrate DeepQR capabilities, we used the commercially available NanoString fluorescent barcodes [6]. These are RNA-specific hybridization probes that report on the identity of the captured RNA target. The probes are designed as linear DNA color barcodes composed of four fluorescent colors arranged in various combinations at six positions along the 6.4 kb M13-DNA template [6] (Figure 1(a)). These barcodes combinatorically generate 4 × 3^5^ = 972 unique detectable color combinations (identical colors at adjacent positions are prohibited to avoid barcodes misidentification). We use tunable spectral imaging (CoCoS) [7] to disperse the barcode emission spatially. Effectively, the linear color barcode is transformed into a two-dimensional QR-code-like image, with color encoded perpendicular to the barcode axis, thus allowing the introduction of additional colors independent of conventional filter channels. In addition, such a configuration allows acquiring all data channels with a single snapshot, reducing acquisition time by a factor of one over the number of colors and eliminating the need for sequential multicolor imaging and chromatic alignment [8].

We benchmarked DeepQR against the current state of the art in single molecule transcriptomics, the NanoString nCounter system. The nCounter gene expression platform is a broadly used optical method for direct single-molecule RNA expression quantification [9]. The four-color barcodes capture RNA targets in solution by hybridization and then extended linearly along the imaging surface. Counting the various barcodes determines the gene expression profile with high accuracy and sensitivity at the single-molecule level. The nCounter system uses four color channels to sequentially acquire the four-color NanoString barcodes (Figure 1(a) top), resulting in 2,220 separate acquisitions per sample. For direct comparison, we designed DeepQR to simultaneously image the four-color barcodes with a single acquisition (Figure 1(b)); therefore, DeepQR requires only 555 acquisitions in order to resolve the same sample. Importantly, we use a single filter with only three-color channels for resolving all four colors, showcasing the ability to resolve two fluorophores in the same spectral window as the basis for the high multiplexing capabilities of DeepQR.

## 2 Results and discussion

DeepQR harnesses the synergistic combination of deep neural networks (DNN) analysis with the continuously controlled spectral-resolution (CoCoS) imaging scheme [7]. In CoCoS, two direct-vision prisms (Figure S1) introduce controlled spectral dispersion in a single axis such that all colors can be imaged simultaneously on a monochrome camera with a single snapshot (Figure 1(a) bottom). Unlike other spectral imaging techniques, where the degree of dispersion is fixed [10]–[15], in CoCoS dispersion may be optimized for specific applications. Tuning the dispersion is crucial for minimizing the spectral footprint of the barcodes, allowing to maximize the density of resolved single molecules in the FOV without introducing overlaps between molecules [8], [11]. Moreover, the simultaneous acquisition with a single emission filter also removes the need for fiducial markers used in nCounter to align the different color channels (Figure S2 and Supplementary Note 1). This frees up ∼9 % of the field of view (FOV), allowing even higher barcode densities and better throughput. DNNs perfectly complement CoCoS as they can recognize even minute spectral changes introduced to the PSF [13], [16], [17], therefore, allowing to minimize further the dispersion required for efficient color classification. The spectrally dispersed images are decoded in DeepQR by a U-Net architecture DNN [18] (see methods and Figure S3) that reconstructs each dispersed FOV into a nondispersed multichannel image.

To demonstrate DeepQR capabilities, we performed a clinical gene expression experiment registering the differential expression signature of patients with ulcerative colitis (UC) using the commercial NanoString inflammation gene expression panel.

**Figure 2:**
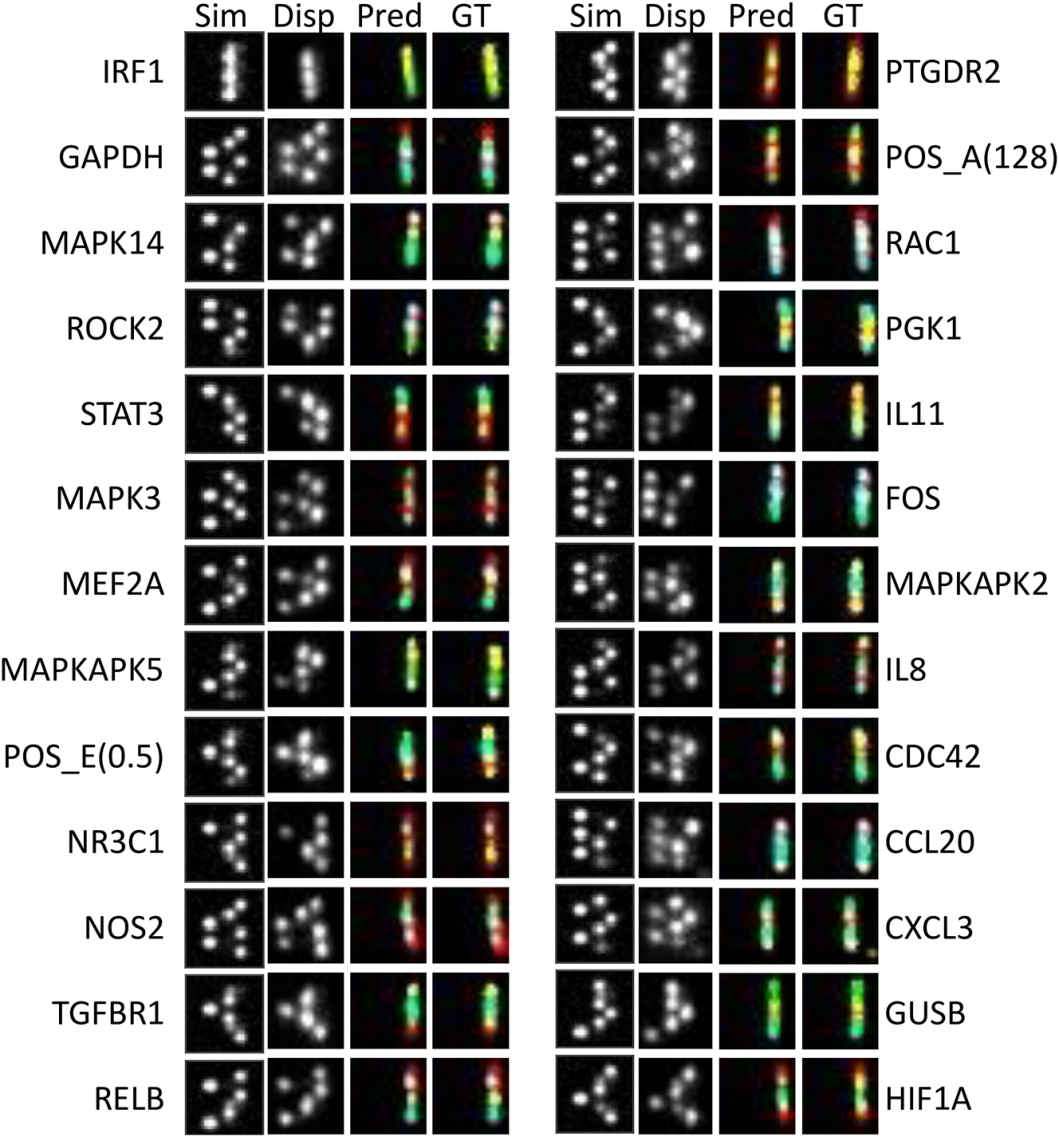
Benchmarking deepQR RNA classification on NanoString’s inflammation panel’s 4-color barcodes. Examples of barcode classification using DeepQR prediction. Leftmost two columns: simulated (Sim) and experimental (Disp) dispersed single frame acquisition excited by all three lasers, capturing the barcode information in a 2D single color barcode. Prediction column (Pred): False color representation of the four-color U-Net prediction, according to the dispersed images. Rightmost column (GT): False colored ground-truth overlays of sequential four-channel acquisition with dedicated laser excitation and emission filter per channel and no dispersion (RPA 180°). The name of the corresponding target gene is shown next to each barcode.

First, we trained the U-Net to convert a single dispersed image to four color channels representing the different fluorophores. For training, we imaged 1,120 FOVs of a single RNA sample tagged with the NanoString barcodes, acquiring for each FOV a dispersed image and corresponding four nondispersed color-filtered images, which were used as our four-channels ground truth. The ground-truth images were acquired by sequentially switching the excitation lasers and emission filters to register each color separately. In contrast, the dispersed images were acquired in a single frame through a multiband emission filter (Figures 1(b) and 2). Our network was trained on 80 % of the dataset minimizing the mean absolute error (MAE) between ground-truth and network predictions (see methods, Figures S4–6 and Supplementary Note 2). The remaining 20 % were used to validate and test the network’s performance on unseen data.

After this training, the same pretrained network was applied to reconstruct the dispersed images of other samples without additional training, resolving NanoString’s four-color barcodes with a single frame instead of four. This results in more than 4-fold faster acquisition speeds (considering it takes ∼50 ms to switch between filters), significantly increasing turnaround times for gene expression analysis, crucial for clinical point-of-care analyses.

Notably, two of the barcode colors, the green Cy3 and yellow Alexa Fluor 594 fluorophores, were spectrally overlapping in the same channel of our system (Figure 1(b)). With DeepQR’s classification, we could resolve them despite their subpixel spectral displacement difference (Figure S7 and Supplementary Note 3). This shows that DeepQR offers better resolution in color classification than that achievable with standard spectral fitting [19], showcasing DeepQR’s ability to resolve more color combinations than filter-based imaging with the same spectral channels (Figure 1(b) and (c)).

**Figure 3:**
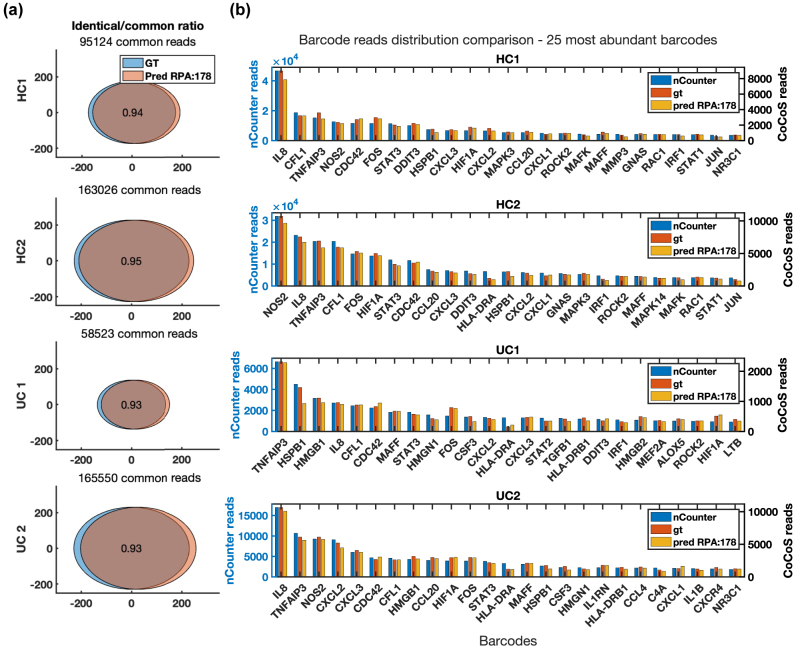
Readout comparison between deepQR prediction (Pred), ground-truth (GT) imaged with CoCoS microscopy, and NanoString’s nCounter system. We compared four samples consisting of two healthy individuals (HC) and two patients with ulcerative colitis (UC) using NanoString’s inflammation barcode panel. (a) A set comparison between matched pairs of barcodes that were eligibly read both in GT and Pred (termed common barcodes). The area of circles corresponds with the number of barcodes in each set (GT or Pred) and the ratio of identical barcodes is presented at the center. (b) Barcode distribution comparison of the 25 most abundant barcodes detected in the three methods.

To evaluate how well DeepQR performed, we analyzed gene expression profiles of four RNA samples obtained from intestinal biopsies of two patients with ulcerative colitis and two healthy individuals (see methods). Previous reports have shown a differential expression pattern in inflammatory genes between healthy and inflamed intestines [20], 21]. We first compared the network’s multichannel predicted output with the multichannel ground truth acquisition (see Supplementary Note 2 and Figures S4–6). Although the MAE between the trained network’s output and the ground truth images was found to be well below the average pixel value, this metric is greatly affected by noise in the FOV and is insufficient to assess the network’s ability to accurately assign barcode colors. Therefore, we evaluated DeepQR’s performance by directly comparing the network-predicted and ground truth barcodes on a barcode-by-barcode basis (Figures 2 and S8). For this purpose, we cropped the same barcode coordinates in both datasets and performed pairwise comparisons. The analysis yielded a 93–95 % concordance between the ground-truth and prediction readouts (Figures 3(a) and S9), demonstrating the effectiveness of our approach despite the suboptimal efficiency of the barcode readout process. This pairwise comparison also enabled us to assess the source of missed or erroneous network classifications (Supplementary Note 4 and Figure S10). One challenge in imaging multiple color markers excited with a single laser is significant bleed-through between emission channels; in our case, the green fluorophore fluoresces also into the yellow emission channel during ground-truth acquisition. We addressed the bleed-through by registering the yellow bleed-through component of the green markers, establishing a global bleed-through correction function to the yellow channel (Figure S11). We note that even barcodes solely composed of yellow and green markers imaged through the same spectral band were correctly classified, such as the one for the IRF1 gene (Figure 2, top left).

In the final step, we compared the gene expression count distributions obtained from our experimental process to those obtained from the nCounter system. We used the cropped barcodes readout to generate global gene-count distributions for both ground-truth and network predicted barcodes. Comparison of DeepQR’s prediction with the results of the standard 4-color imaging of both ground-truth and nCounter revealed a good alignment of the raw gene count distributions (Figures 3(b) and S12).

**Figure 4:**
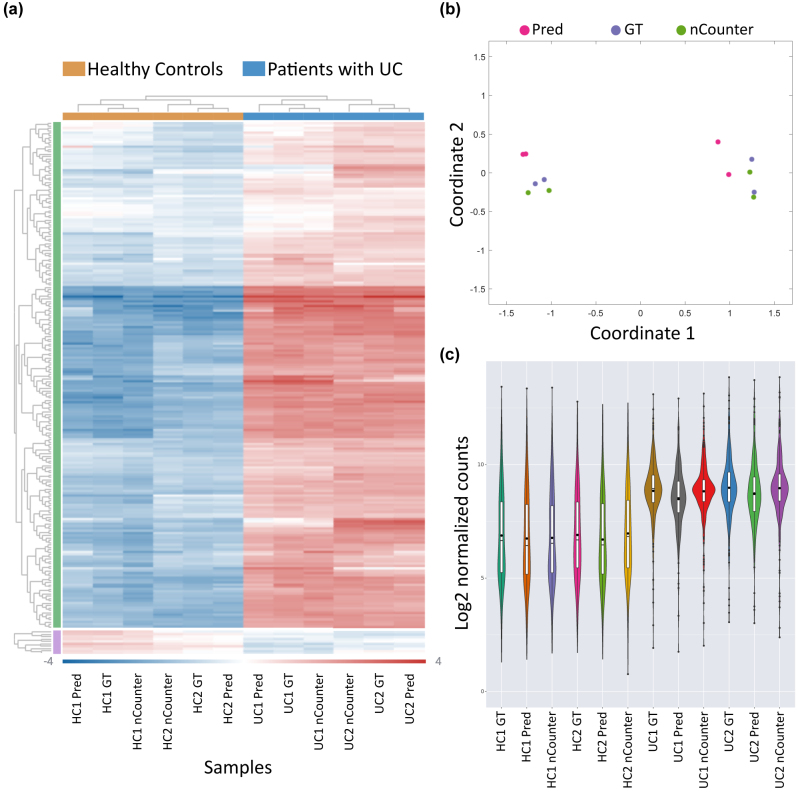
Gene expression analysis using DeepQR for ulcerative colitis detection. (a) Unsupervised clustering and heatmap representation of the mean-subtracted normalized log2 barcode expression values, comparing four samples of healthy individuals and patients with ulcerative colitis (UC) between the three detection methods: DeepQR prediction (Pred), ground-truth (GT), and the commercial NanoString nCounter system. (b) Multidimensional scaling (MDS) plot showing the classification of healthy and UC samples between the three methods. (c) Log2 of normalized barcode count distributions across all samples and readout methods.

To determine whether the barcode-prediction readout is clinically applicable and sufficiently accurate for sample classification, we normalized the barcode distribution according to the standard protocols used in NanoString gene expression analysis pipeline (via ROSALIND^®^ interface, as described in methods). The results presented in Figure 4 demonstrate that the DeepQR method can accurately reconstruct the normalized gene expression distributions of the four samples and correctly classify ulcerative colitis patients in an unsupervised manner.

## 3 Conclusions

In conclusion, we demonstrate a novel approach for fast and efficient color registration and multiplexing at the single-molecule level. DeepQR is compatible with demanding multicolor single-molecule applications, enabling immediate utilization in applications requiring high multiplexing capabilities. DeepQR provides a traditional multichannel output compatible with standard downstream analyses and supports multiplexing of spectrally overlapping colors while completing the entire color acquisition pipeline without exchanging filters and at a fraction of the standard acquisition time.

Specifically, we demonstrated that DeepQR resolves four-color barcodes with 93–95 % accuracy using only three spectral channels with two of the four colors spectrally overlapped and excited with the same laser. This resolution provides 972 distinctly resolvable barcodes compared to 96 barcodes with conventional filter-based three-channel microscopy. Consequently, this could be used for addressing higher degree multiplexing sorely needed in single-molecule transcriptome analysis. DeepQR recorded the NanoString inflammation panel in less than a quarter of the standard acquisition time and without any fiducial markers, achieving results highly correlated with the nCounter system. Beyond the similarity in gene count distributions, the majority of differentially expressed genes were identical in both methods (Figures S13 and 14, Table S1 and Supplementary Note 5), which emphasizes the potential for expanding the gene panel accessible by optical imaging and enabling differential gene discovery for clinical purposes.

## 4 Methods

### 4.1 Sample preparation

Intestinal biopsies were obtained from two patients with ulcerative colitis undergoing routine colonoscopies and two healthy individuals as a control. Biopsies were immediately transferred to the laboratory in complete medium (CM), consisting of RPMI 1640 supplemented with 10 % fetal calf serum (FBS) 100 U/ml penicillin, 100 μg/ml streptomycin, and 2.5 μg/ml amphotericin B (Fungizone) on ice (to preserve the intact tissue alive). Samples were then washed with sterile phosphate buffer saline (PBS) (Biological Industries) and cultured in CM supplemented with 100 μg/ml gentamicin (Biological Industries) and 0.001 % DMSO (Sigma-Aldrich) in an atmosphere containing 5 % CO2 at 37 °C for 18 h.

For RNA extraction, biopsies were homogenized in ZR BashingBead Lysis Tube (Zymo Research) using a high-speed bead beater (OMNI Bead Ruptor 24). Total RNA was extracted using Trizol^®^ (Invitrogen) according to a standard protocol. RNA concentration and quality were assessed using NanoDrop Spectrophotometer (Thermo Scientific). The 260/280 and 260/230 ratios in all samples were >1.8.

### 4.2 NanoString barcode hybridization and readout

Two cartridges, each containing the same four samples, were prepared according to the manufacturer’s instructions using the nCounter Human Inflammation V2 Panel (NanoString). Briefly, hybridization buffer combined with the codeset of interest is combined with 5 μl (400 ng) of total RNA and incubated at 65 °C overnight. To remove all fiducial markers for imaging on the CoCoS system, we extracted all the imaging buffer containing the fiducial markers from one of the reagent plates prior to loading it onto the prep station. Samples were then loaded onto the prep station and incubated under high sensitivity program for 3 h. Following the prep station, one cartridge was read using NanoString digital analyzer with the high-resolution option, while the other was loaded with imaging buffer that did not contain the added fiducial-marker beads and was read using DeepQR. The raw barcode counts were output both by DeepQR and digital analyzer in RCC files and were further normalized and processed by the same analysis pipeline as described below.

### 4.3 Optical setup

The optical setup was primarily equivalent to the one introduced previously in ref. [7], with minor changes in the choice of the emission telescope’s lenses (see schematic system sketch in Figure S1).

#### 4.4.1 Excitation

For excitation, we used three lasers (Cobolt AB, Sweden) with wavelengths 488 nm (MLD 488, 200 mW max power), 561 nm (Jive 561, 500 mW max power), and 638 nm (MLD 638, 140 mW max power). All lasers were mounted on an in-house designed heatsink, which coarse aligned their beam heights. Each laser beam was passed through a clean-up filter (LL01-488-12.5, LL01-561-12.5, LL01-638-12.5, Semrock, USA) and expanded to 12.5–20× its original diameter (3×LB1157-A, 3×LB1437-A, Thorlabs, USA). A motorized shutter (SH05, Thorlabs, USA) was used for modulating on/off the solid-state 561 nm laser, while the diode lasers were modulated directly on the laser head. The beams were then combined into a single beam using long-pass filters (Di03-R488-t1-25.4D, Di03-R561-t1-25.4D, Semrock, USA). To homogenize the excitation profile of the sample, the combined beam was passed through an identical setup to the one described in the work of Douglass et al. [22]. In short, the combined beam was injected into a compressing telescope (AC254-150-A-ML, AC254-050-A-ML, Thorlabs, USA) with a rotating diffuser (24-00066, Süss MicroOptics SA, Switzerland) placed ∼5 mm before the shared focal points of the telescope lenses (Figure S1). A series of 6 silver mirrors (PF10-03-P01, Thorlabs, USA) was then used to align the beam into a modified microscope frame (IX81, Olympus, Japan), through two identical microlens arrays (2×MLA, 18-00201, Süss MicroOptics SA, Switzerland) separated by a distance equal to the microlenses focal length and placed inside the microscope frame. The homogenized beam was reflected onto the objective lens (UPlanXApo 60× NA1.42, Olympus, Japan) by a four-band-multichroic mirror (Di03-R405/488/561/635, Semrock, USA). The sample was placed on top of motorized XYZ stage (MS-2000, ASI, USA) with an 890 nm light-emitting diode (LED)-based autofocus system (CRISP, ASI, USA), which enabled scanning through multiple fields of view.

#### 4.4.2 Emission

The emitted fluorescence light was gathered by the same objective and transmitted through the multichroic mirror onto a standard Olympus tube lens to create an intermediate image at the exit of the microscope frame. This image was passed through a filter wheel (Sutter Lambda 10-B, Sutter Instruments, USA) with three emission filters: multiband filter (FF01-440/521/607/694/809-25, Semrock, USA), 575/15 (FF01-575/15-25, Semrock, USA), or 620/14 (FF01-620/14-25, Semrock, USA). Light was then directed into a magnifying telescope (Apo-Rodagon-N 105 mm, Qioptiq GmbH, Germany and Olympus’ wide field tube lens with 180 mm focal length, #36–401, Edmund Optics, USA), with two commercial direct vision prisms (117240, Equascience, France) placed within the infinity space between the lenses and mounted on two motorized rotators (8MR190-2-28, Altechna UAB, Lithuania) controlling the prisms’ angles around the optical axis. The final image was acquired on a back illuminated sCMOS camera (Prime BSI, Teledyne Photometrics, USA).

Image acquisition was coordinated using the micromanager software [23], controlling camera acquisition, laser excitation, *XY* stage location, and prism rotator angles. The camera and lasers excitation were synchronized using an in-house built TTL controller based on an Arduino^®^ Uno board (Arduino AG, Italy) [24].

### 4.4 Image acquisition

Each of the four sample lanes was fully scanned laterally and imaged, obtaining approximately ∼1,000 FOV per sample lane. In each one of the FOVs, a six-image acquisition was taken with specifications according to Table 1:

**Table 1:** Image acquisition parameters. Consecutive frames were acquired according to the order listed in the table using the same laser intensities and camera exposure.

Excitation	RPA	Emission filter	Exposure time	Laser intensities
All lasers simultaneously	178	Multiband filter	300 ms	638 laser: 0.28 kW/cm^2^
All lasers simultaneously	180	Multiband filter		561 laser: 0.33 kW/cm^2^
638 laser	180	Multiband filter		488 laser: 0.3 kW/cm^2^
561 laser	180	620/14 (AF594)		
488 laser	180	Multiband filter		
561 laser	180	575/15 (cy3)		

The full lane acquisitions were stacked in FIJI [25], resulting in a multi-FOV hyperstack with six channels that were input to the deep learning (DL) analysis pipeline.

### 4.5 NanoString nCounter image acquisition

The nCounter experimental assay has been thoroughly described previously [6] and is given here for comparison completeness. Briefly, the barcoded samples mixed with Tetra-speck microspheres (used as fiducial markers) are stretched and immobilized on specialized slides. The slides are scanned and each FOV is imaged four times with different excitations and emission filters (Figure 1(a)) to detect the four-colored barcodes sequentially. The Tetra-speck microspheres are then used for image registration of the four different color channels and chromatic aberration correction (Figure S2).

### 4.6 Deep learning analysis

Converting the dispersed image stacks to nondispersed, 4-channels, multi-FOV hyperstacks was implemented with DL using Tensor-flow and Open-CV packages in Python. The full code is available for download from the Ebenstein lab’s GitHub (https://github.com/ebensteinLab). The DL architecture used was based on a U-Net architecture [18], a fully convolutional encoder-decoder neural network. This architecture is indifferent to the dimensions of the input image and uses skip connections between the encoder and decoder parts of the network to preserve spatial features encoded in different levels and may have been lost in the encoding process (see Figure S3 for architecture’s scheme). Each layer consists of two sets of 3 × 3 convolution filters and nonlinear activation layers, succeeded by a 2 × 2 down-sampling or up-sampling operation for the encoder and decoder parts, respectively. Due to our dispersion-to-color conversion task, we adjusted the original U-Net architecture such that the dimensions of the output image were changed to four channels.

To train the DNN model, we used a full sample lane hyperstack, consisting of 1,120 FOVs acquired with the same parameters as in Table 1. The network’s input was 1,120, single-channel, dispersed FOVs paired with 1120 four-channel FOVs as ground-truth. To artificially increase the data for training, each 1,024 × 1,024 pixels^2^ FOV was segmented into 49 overlapping crops of 256 × 256 pixels^2^ with a 50 % overlap between adjacent crops. This dataset was divided into 80 % training data, 10 % validation data, and 10 % test data and was trained over 200 epochs setting the model’s weights to minimize the mean absolute error (MAE) loss (Figure S4). The final model’s weights were saved according to the minimal validation loss. To further refine the trained model and improve the distinction between the green (Cy3) and yellow (AF594) fluorophores, we retrained the network with the same dispersed input paired with the green ground-truth channel only. This produced a second, more accurate model for the green channel. We then used the first model to predict the red (AF647), yellow (AF594), and blue (AF488) channels and the second to predict the green (Cy3) channel and combined their results to create the output four-channel hyperstack. Since our model was trained on 256 × 256 pixels^2^ patches, for prediction, we divided the 1,024 × 1,024 pixels^2^ dispersed FOVs input into 16 patches of 256 × 256 pixels^2^. After prediction, we stitched the predicted patches to enable visual comparison of the full FOVs (Figure S15). Finally, we used the two models trained on this sample lane to predict the results of the other four sample lanes without additional training, allowing us to extract the full distribution of barcodes from each sample.

To assess the amount of data needed to achieve optimal results, we evaluated our training procedure over smaller subsets of randomly selected FOVs showing the tradeoffs of training on smaller datasets and reducing the number of training epochs (see Figures S5 and S6 for results). To avoid a nonrepresentative validation subset in training small subsets of FOVs, we first filtered out any out-of-focus or noisy FOVs by applying a set of criteria to the dispersed FOV. Any FOV with mean values >600 analog-to-digital units (ADU), pixels standard deviation values outside 100–600 ADU, or maximal pixel value <3,000 ADU was discarded.

### 4.7 Image analysis

FOV filtering: Prior to barcodes readout from the hyperstacks, we first removed out-of-focus or noisy FOVs to avoid false barcode readouts. The filtration procedure was carried out in FIJI using the ground-truth hyperstacks as follows:

1. Sum all four hyperstack color channels to produce a grayscale multi-FOV stack.
2. For each FOV measure, the mean and standard deviation of all pixel values.
3. Filter out FOVs with mean values outside the 500–700 ADU range or standard deviations outside the 50–1,000 range.

Barcode detection and cropping: To reliably compare the barcode readout between the ground-truth and prediction hyperstacks, we wanted to compare readouts from the same locations in both hyperstacks. Therefore, we detected the barcodes on the ground-truth hyperstack by applying the multi-template matching plugin in FIJI [26] on the color-channel-summed ground-truth stack (see Figure S8 for example workflow). This allowed us to localize all barcode-like features in the ground-truth stack and to extract a 20 by 9 pixels crop from each of these locations both in the ground-truth and prediction 4-channel hyperstacks. The procedure was carried out as follows:

1. Sum all four hyperstack color channels to produce a grayscale multi-FOV stack.
2. Apply the Multi-Template Matching plugin with a cropped grayscale barcode template (complete parameter list is provided in Figure S8).
3. Split the color channels of both ground-truth and prediction hyperstacks.
4. Use FIJI ROI manager Multi-crop function to crop the same barcode detections from all channels of both ground-truth and prediction hyperstacks.
5. Merge the cropped barcode stacks channels to receive multi-barcode hyperstacks, allowing a location-based comparison between barcodes.

### 4.8 Bleed-through correction and barcode readout

One major issue we had to overcome in resolving the correct color sequence of the barcodes was the bleed-through from the green (Cy3) channel to the yellow (AF594) channel. Due to the spectral properties of these fluorophores and our excitation wavelength, an emission filter-based separation of the two fluorophores was insufficient, and postacquisition correction of the barcode images was employed (see Figure S7). To readout the barcodes color sequence from the cropped barcode stacks, we used a custom readout Matlab code that follows these steps:

1. Import the barcode image stacks using built-in Matlab functions for tiff file reading.
2. Create profiles along both barcode axes to extract initial peaks locations in all channels using the built-in Matlab function findpeaks.
3. Peaks along the barcode axis that are wider than the nominal PSF were fitted by a two-Gaussians model to resolve overlapping PSFs (which might occur due to small focus deviations).
4. Localize the peaks in the red (AF647), green (Cy3), and blue (AF488) channels by 2-d Gaussian fits at the initial positions using FastPsfFitting Matlab functions written by Simon Christoph Stein and Jan Thiart, which are available on Matlab file exchange. The peak localization of the yellow (AF594) channel is done after bleed-through correction.
5. To characterize the bleed-through from the green to the yellow channel, find all complete barcodes containing six localizations without the yellow markers and at least one localization in the green channel.
6. Estimate the mean parameters for the bleed-through PSF according to the green marker PSF: x-shift, y-shift, intensity ratio, and standard deviation ratio.
7. Use these parameters to subtract simulated bleed-through PSFs from the yellow channel images according to the green channel localization results and then localize the yellow markers on the bleed-through corrected images.
8. Combine all channels readout and perform quality check (QC) according to known barcode limitations: six markers per barcode, adjacent markers should have different colors, minimal separation between adjacent markers along the barcode’s axis, and maximal shift between markers along the perpendicular axis. Barcodes that did not meet the QC limitations were discarded.
9. The final barcode readout is then organized in a table and enumerated according to its crop number in the barcodes crop stack for the following ground-truth to prediction barcode readout comparison.

### 4.9 Barcode to gene counts conversion

After reading the color code of the barcodes, a conversion to the corresponding gene names was performed using the RLF file provided with the nCounter dataset, containing the code-set conversion between color-code and gene identity.

The total barcode reads of each barcode were counted in the ground-truth and prediction tables and assigned to their relevant genes according to the RLF. Only barcode reads corresponding to the nCounter code-set were kept.

### 4.10 ROSALIND^®^ NanoString gene expression

For creating the gene expression heatmap (Figure 4(a)), sample MDS plots (Figure 4(b)), and violin plots (Figure 4(c)), data were analyzed by ROSALIND^®^ (https://rosalind.bio/), with a HyperScale architecture developed by ROSALIND, Inc. (San Diego, CA). Normalization, fold changes and *p*-values were calculated using criteria provided by NanoString. ROSALIND^®^ follows the nCounter^®^ Advanced Analysis protocol of dividing counts within a lane by the geometric mean of the normalizer probes from the same lane. Housekeeping probes to be used for normalization are selected based on the geNorm algorithm as implemented in the NormqPCR R library [27]. Fold changes and *p*Values are calculated using the fast method as described in the nCounter^®^ Advanced Analysis 2.0 User Manual. *P*-value adjustment is performed using the Benjamini–Hochberg method of estimating false discovery rates (FDR). Clustering of genes for the final heatmap of differentially expressed genes was done using the PAM (Partitioning Around Medoids) method using the fpc R library [28] that takes into consideration the direction and type of all signals on a pathway, the position, role and type of every gene, etc. To effectively compare the same four samples across the three analysis methods (GT, Prediction, and NanoString’s nCounter), we used Rosalind’s covariate correction analysis with the detection method as a hidden covariate. The uncorrected gene expression heatmap is presented in Figure S18.

### 4.11 Heatmap analysis

The two-color heatmap (Figures 4(a) and S18) represents the mean-subtracted normalized log2 expression values, i.e., for each gene, the average of the log2 normalized expression is taken and subtracted from each sample’s expression.

### 4.12 Barcode detection performance analysis

Ground-truth and prediction barcode detection performances were compared to one another and to nCounter readout of the same samples in a different experimental run.

Ground-truth versus prediction comparison

To compare barcode detection performance between ground-truth and prediction, we first filtered only the “common barcode reads” where both the ground-truth and prediction obtained valid barcode detection (barcodes that passed our filtering QC). Out of these common valid reads, we compared each barcode readout and counted the number of identical reads in both stacks (Figures 3(a) and S9). The Venn diagram representation of the ratio of identical barcodes out of all common barcodes was generated using Darik Gamble’s “venn” Matlab script available online from Matlab Central file exchange.

Histogram comparison of raw barcode counts

To compare the nCounter results to our readout, all RCC files obtained from the NanoString digital analyzer were first exported to csv files using the nSolver 4.0 software. The barcodes obtained from our readout were counted according to their color sequence using the built-in Matlab function “histcounts.” Finally, to assess our readout pipeline, the raw barcode counts from the nCounter were compared to our readout from the ground-truth and prediction stacks by plotting the 25 most abundant endogenous genes in histograms (Figure 3(b)).

### 4.13 PSFs simulations

All simulations were performed by a custom Matlab code (code is provided as Supplementary Material). Here, we provide a short description of the pipeline:

1. Excitation and emission spectra of 4 commercial fluorophores together with our 5-band emission filters (FF01-440/521/607/694/809-25, Semrock, USA) for the 4-color barcodes simulations were downloaded from Semrock’s SearchLight™ spectra viewer. Each of the fluorophores’ spectrum was multiplied by the filter’s spectrum to produce the actual spectrum imaged on our camera.
2. The wavelength to pixels displacement calibration curve of our CoCoS setup (which was calculated previously [7]) was adjusted according to the experimentally used RPA by multiplying the entire curve by sin((180-RPA)/2).
3. Barcodes fluorophore combinations were then simulated by converting each fluorophore’s spectrum into a diffraction-limited dispersed image. This was done by assigning a Gaussian with unity amplitude and 1.2-pixel standard deviation to each wavelength in the emission spectrum. Each Gaussian was displaced according to the RPA-adjusted displacement curve and summed together with other Gaussians. Finally, the total summed intensity of all Gaussians was normalized to unity and multiplied by an excitation efficiency factor, which was calculated by the excitation spectrum value (fractions only) at the excitation laser wavelength.
4. This process was repeated for the randomly selected fluorophores at the six barcode locations, and all images were summed to provide the barcode’s spectral image.
5. To further resemble the experimental images, a noise model was added to all simulated spectral PSF images using the imnoise function in Matlab. The noise model used in this work was a sum of a Poisson distributed shot-noise and Gaussian noise with a constant mean of 0.3 and 0.000625 variance.

## Supporting Information

Additional experimental details and methods are provided in the supporting information file (PDF).

## Supplementary Material

Supplementary Material Details
