# Supplementary material for: DeepQR: single-molecule QR codes for optical gene-expression analysis
Source: Nanophotonics. 2024 Jul 30;14(15):2549–61. doi: 10.1515/nanoph-2024-0236 (PMC12322721; doi:10.1515/nanoph-2024-0236)
Supplement: Supplementary file 1 — Supplementary Material Details [file j_nanoph-2024-0236_suppl_001.pdf]

Supplementary Information:

DeepQR: Single-molecule QR codes provide extreme multiplexing for optical gene-expression analysis

Jonathan Jeffet[1,2,4], Barak Hadad[4,5], Sahar Froim[4,5], Kawsar Kaboub[6,7], Keren M. Rabinowitz[6,7], Jasline Deek[2], Sapir Margalit[2], Iris Dotan[6,7], Alon Bahabad[4,5], Yuval Ebenstein[2,3,4] *

1. School of Physics and Astronomy, the Raymond and Beverly Sackler Faculty of Exact Sciences, Tel Aviv University, Tel Aviv 6997801, Israel
2. School of Chemistry, the Raymond and Beverly Sackler Faculty of Exact Sciences, Tel Aviv University, Tel Aviv 6997801, Israel
3. Department of Biomedical Engineering, Fleischman Faculty of Engineering, Tel Aviv University, Tel Aviv 6997801, Israel
4. Center for Light Matter Interaction, Tel Aviv University, Tel Aviv 6997801, Israel
5. Department of Physical Electronics, School of Electrical Engineering, Fleischman Faculty of Engineering, Tel-Aviv University, Tel-Aviv 6997801, Israel
6. Division of Gastroenterology, Rabin Medical Center, Petah Tikva, Israel
7. Felsenstein Medical Research Center, Sackler Faculty of Medicine, Tel-Aviv University, Tel Aviv, Israel

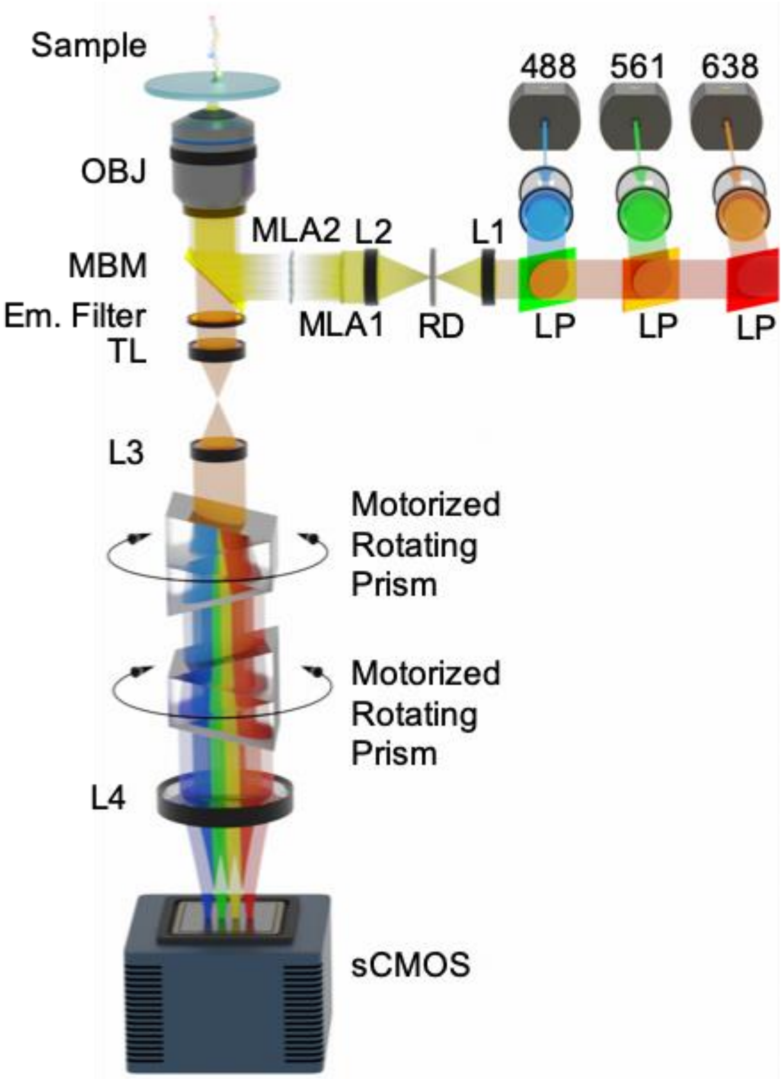

Figure S 1. Optical scheme (full description in the methods section). Abbreviations: LP, Long pass filter; L1-4, lens; RD, rotating diffuser; MLA1-2, micro-lens array; MBM, multi-band mirror; OBJ, objective; Em. Filter, emission filter; TL, tube lens; sCMOS, scientific complementary metal-oxide semiconductor camera.

Supplementary note 1: **Fiducial markers excluded area calculation**

If a barcode overlaps with a fiducial marker, it will not be read correctly in the NanoString pipeline and will be discarded. To quantify the percentage of inaccessible FOV due to fiducial markers in the standard NanoString pipeline, we used ten non-dispersed FOV simultaneously excited by all three lasers (example FOV in figure S2). We used an intensity threshold to locate all fiducials and created a fiducial binary mask using FIJI's [1] IsoData stack to binary conversion. As each barcode takes up an area of ~5✕15 pixels², we convolved the binary fiducial mask with a 5✕15 all-ones matrix to produce a binary estimate of the excluded area (figure S2, right panel). The percentage of FOV excluded by fiducial markers was calculated as the mean value over an area of 600✕600 pixels² at the center of ten convolved binary images resulting in 9.0 ± 1.1%.

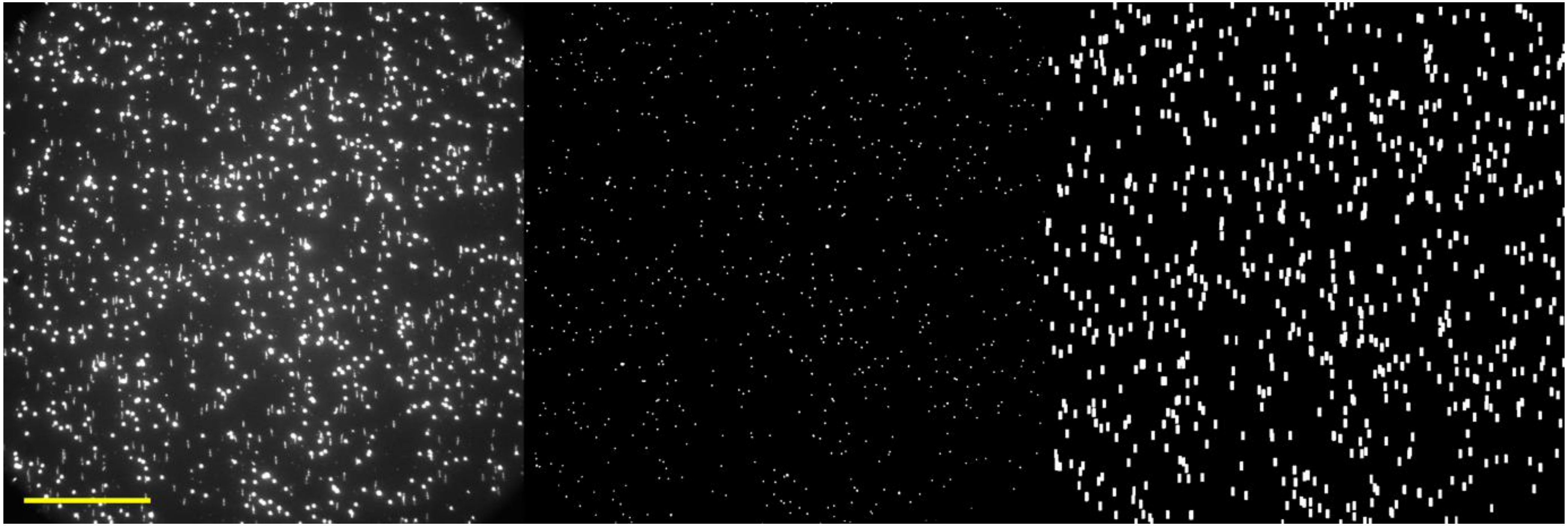

Figure S 2. Fiducial markers impact on the standard NanoString analysis' throughput. Left, a standard NanoString sample imaged without dispersion (RPA=180°) on CoCoS excited by all excitation lasers. The beads appear as brighter, symmetric spots, while the NanoString barcodes appear as extended lines. Middle, a binary mask produced by FIJI according to IsoData thresholding, containing all fiducial markers locations. Right, excluded area created by the fiducials where barcodes will overlap with the fiducials and, therefore, will be discarded from the analysis. The overall fiducials induced excluded area was calculated to be 9.0±1.1% over a subset of ten representative FOVs. Scale bar: 30µm.

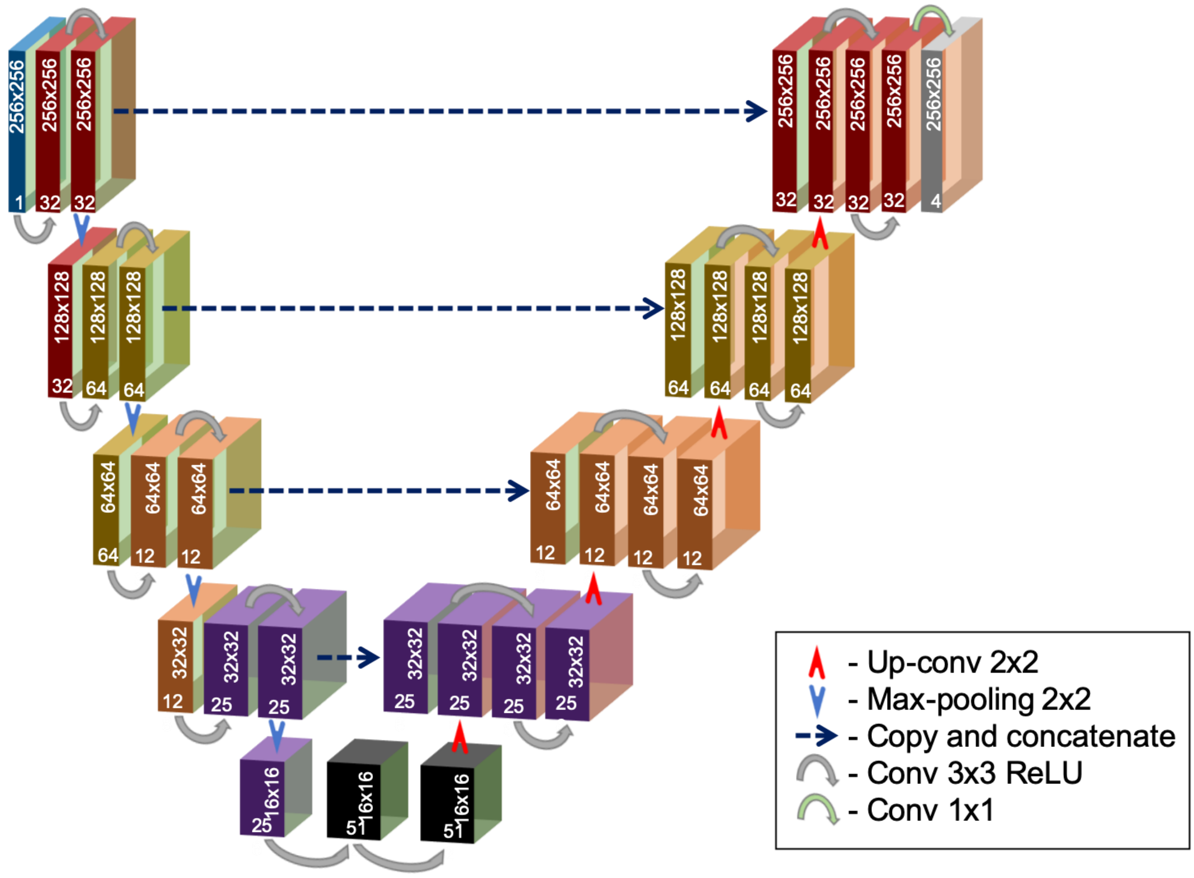

Figure S 3. Neural network model architecture based on the U-NET architecture. Each box corresponds to a set of different feature maps. The colors of the boxes indicate the number of channels at the output of each layer; this number is also displayed at the bottom of each box. The width and height of each layer's output are given at the side of the box. Each arrow indicates a unique type of operation, as displayed in the legend. The red and blue arrows represent up-convolution or max-pooling operations, respectively, that change (reducing or increasing respectively) the feature maps' spatial size. The blue dashed arrows illustrate a copy and concatenate operation, where we take feature maps from the encoder side and add them to the decoder side. The gray and green arrows represent different convolution operations with different kernel sizes, where an additional ReLU activation is added at the output of the layer of a gray arrow operation. The output and input channels dimensions are set to a single channel at the input and translated to four channels at the output.

Supplementary note 2: **U-Net Training Procedure**

As explained in the methods, to train our deep neural network (DNN), we minimized the loss function, i.e. the mean absolute error (MAE) between the predicted and GT images. Figures S4-6 show the evolution of this training by the history of the loss function and the mean squared error (MSE) metric scores. We divided the training into two separate models, to better characterize the minute differences between the green and yellow PSFs in the dispersed images. First, we trained a model converting each dispersed image into three channels: red, yellow and blue (RYB). This was followed by another training of the same network to output a second model extracting only the green (G) channel from the same dispersed images.

Using the ADAM optimizer [2] to optimize the weights of the U-Net model, the loss was minimized in an iterative procedure over 200 epochs. At the end of each epoch, the final weights were applied to predict the validation subset and evaluate the corresponding loss score. The weights which obtained the minimal validation loss score over the 200 epochs of training were saved as the optimal model.

Figures S4-6 capture this training process, displaying the MAE and MSE metrics scores along the training process. The vertical lines in these figures correspond with the epoch where the model obtained the minimal metric value for each model (RYB or G) over the validation subset. The dotted lines show the minimal metric score value providing a guide for the eye to the convergence of the training.

In figure S4, our models were trained over 200 epochs on a full sample lane consisting 1120 FOVs, of which 80% were used for training (blue and yellow solid lines), 10% for validation (orange and purple solid lines), and 10% for testing (scores displayed on subplots' titles). The training, validation and test subset were randomly selected from the entire dataset. In the figure, the validation loss scores for both models seemingly converge after approximately 100 epochs with apparent overfitting (the training scores are lower than the validation scores). However, as seen by the vertical lines, the minimal loss values for the validation subset are obtained at the end of the training after ~200 epochs.

To evaluate the amount of data needed to be recorded for the training, we trained the same networks with smaller subsets of randomly selected in-focus FOVs (a total of 1018 FOVs after filtering). Of these 1018 FOVs, 102 were allocated for a comparative test of all subset-trained models. To provide realistic training, 80% of each subset's FOVs were allocated for training and 10% for validation. Figures S5-6 show the training evolution of these models' loss scores (figure S5) and MSE metric scores (figure S6). Comparing the scores obtained over the same test subset allows us to objectively compare the models' performance, showing a clear advantage for training over larger datasets as expected.

For a reference to the obtained MAE and MSE values, the mean pixel value across the entire RYB dataset (1120 FOVs) was 162±67 ADU, and in the G dataset 120±54 ADU.

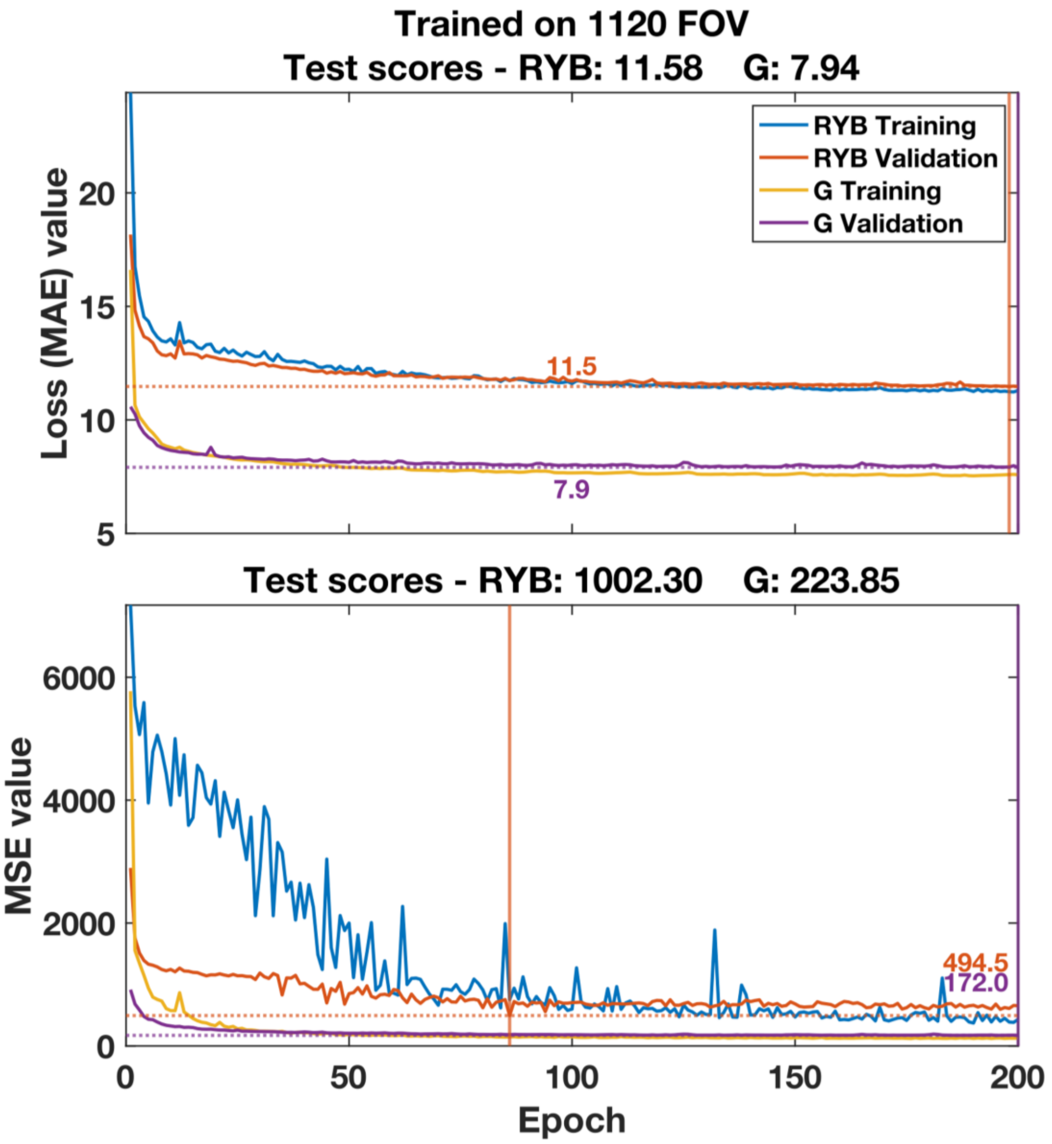

Figure S 4. DNN model training over a full sample lane. Top, loss scores evolution with training for the red, yellow, blue (RYB) and green (G) models. Vertical lines mark the epoch where a minimal validation score was achieved for each model (RYB-orange, G-purple). Best MAE validation scores are illustrated by the dashed lines with their corresponding values. The test scores of the best model (with minimal validation loss score) are shown in the subplot title. Bottom, MSE metric values evolution (not optimized by the model) vertical lines correspond with the best MSE values achieved on the validation set. The test MSE scores of the best model (with minimal validation MAE score) are shown in the subplot title.

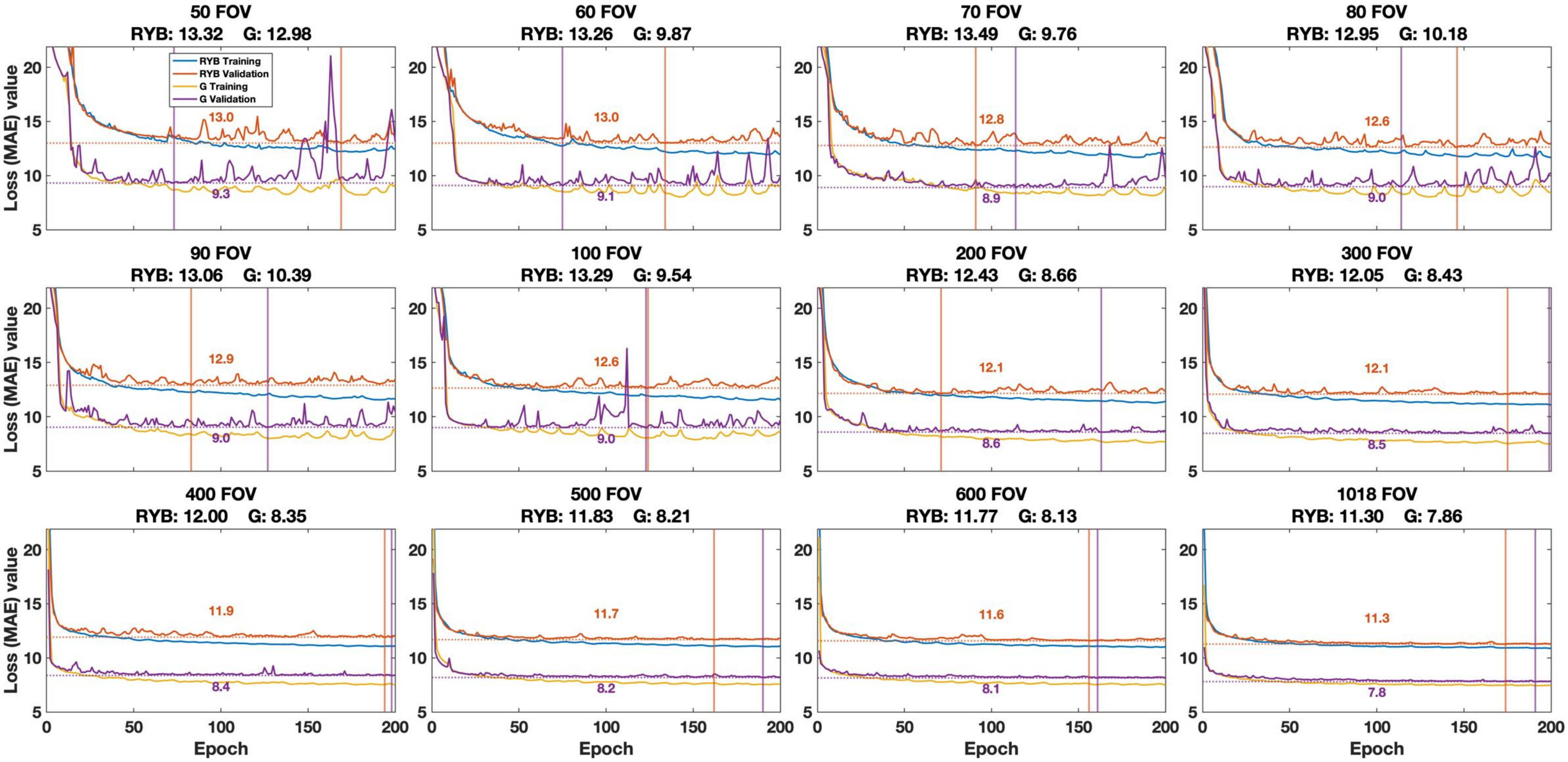

Figure S 5. DNN model training on subsets of filtered FOVs. The total number of FOV used for training and validation is given at the title of each sublot together with the MAE score for the performance of each network on the same test subset (102 independent FOV). Each of the plots depicts the training and validation loss (MAE) history per epoch of the red-yellow-blue (RYB) and green (G) models, which were trained consecutively. Dashed horizontal lines correspond to the minimal validation value of each of the RYB and G networks. Vertical lines pinpoint the epoch with minimal validation score.

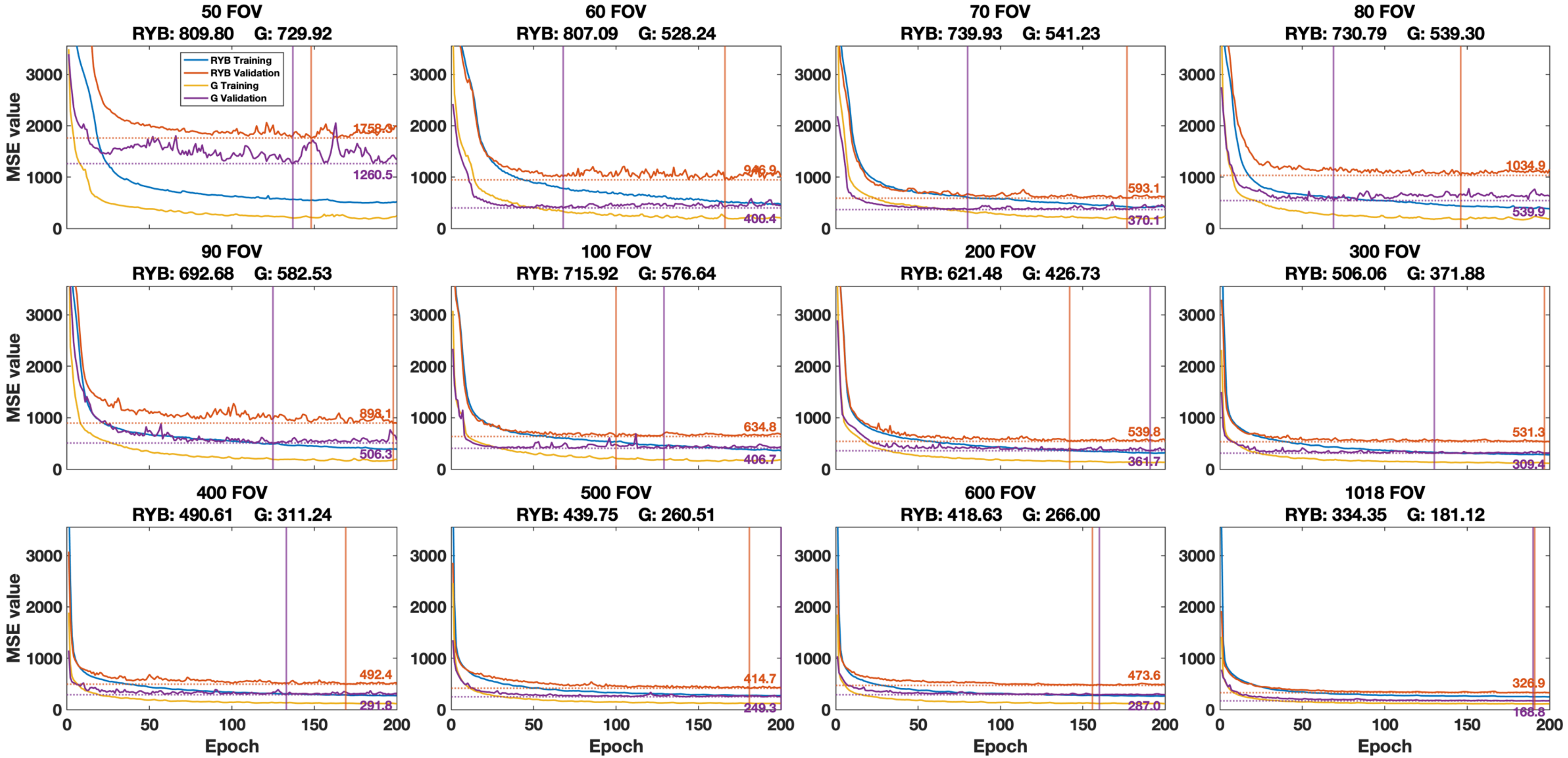

Figure S 6. Same as the previous figure but for MSE metric values (models optimized with MAE and not MSE).

Supplementary note 3: **Weighted spectral centroid calculation**

The spectral difference between the Cy3 and AF594 emissions, presented in figure S7, was calculated using the weighted centroid of each color's intensity profile. To calculate the weighted centroids, we first extracted intensity profiles for each marker in all spectral dispersions by averaging the intensity along a three pixel-wide line centered around each marker for all dispersions. Then we subtracted the background value from each profile and calculated the weighted centroid by the following equation:

$$WC = \frac{\sum x_i \cdot I_i}{\sum I_i}$$

Where WC stands for weighted centroid in pixels, x_i is the pixel location along the line profile, and I_i is the intensity at the i-th pixel. Finally, each of the markers' profiles was aligned to the weighted centroid locations in their respective no dispersion profile (RPA=180°), setting them as the reference location for spectral displacement for each marker.

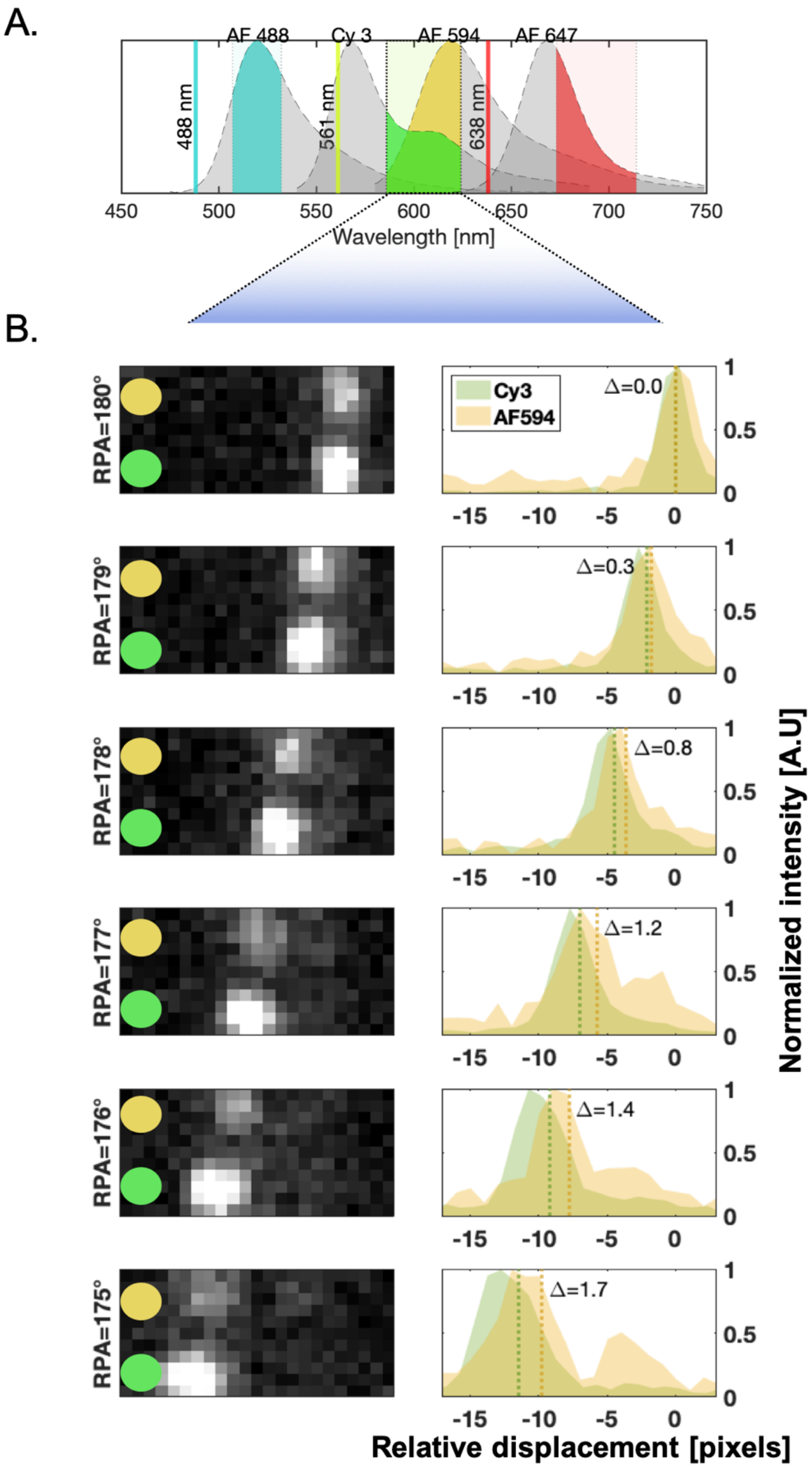

Figure S 7. Resolving Cy3 and AF594 barcode markers imaged through the same spectral channel. A) The emission spectra of the four dyes overlaid with the emission transmission output of the optical system with multi-band filter (same as figure 1B). B) Changing the relative prism angle (RPA) between the two direct-vision prisms controls the dispersion, increasing the spectral resolution as RPA decreases from no dispersion at RPA=180° (top). On the left, crops of two markers from the same barcode excited with 561nm laser at different dispersions. On the right the corresponding normalized intensity profiles of the markers plotted relative to the no dispersion (RPA=180°) weighted centroid location. Dotted lines correspond to the weighted spectral centroid calculated for each profile; delta represent the difference between centroids in pixels.

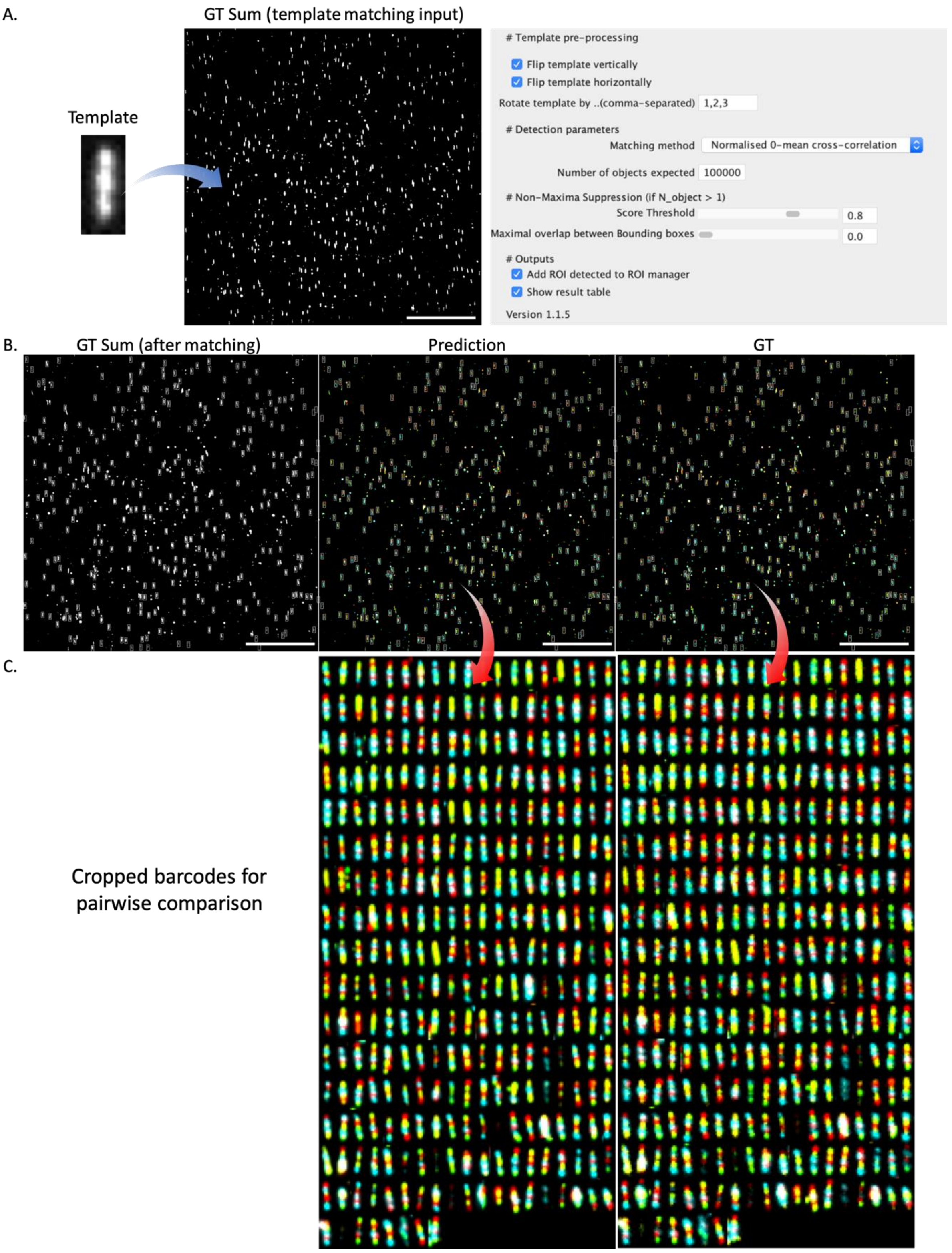

Figure S 8. Template matching barcode extraction. A) As explained in the methods section, the barcodes were detected and cropped using the multi-template matching plugin in FIJI. The ground-truth (GT) four-channel multi-FOV hyperstacks were summed over all channels to create a grayscale multi-FOV stacks. The barcode shown to the left was used as a template to detect all barcode instances in the multi-FOV stacks with the same plugin parameters for all GT sample stacks. B) After matching, the same regions of interest (ROIs) that were detected on the summed GT stack (left) were cropped from the four-channel multi-FOV hyperstacks of U-Net prediction (center) and GT (right). All scale bars equal 30 µm C) The barcode crops (here shown as a montage of all the barcodes cropped from the above example FOV) were paired to enable pairwise comparison in the following analyses as described in the methods section.

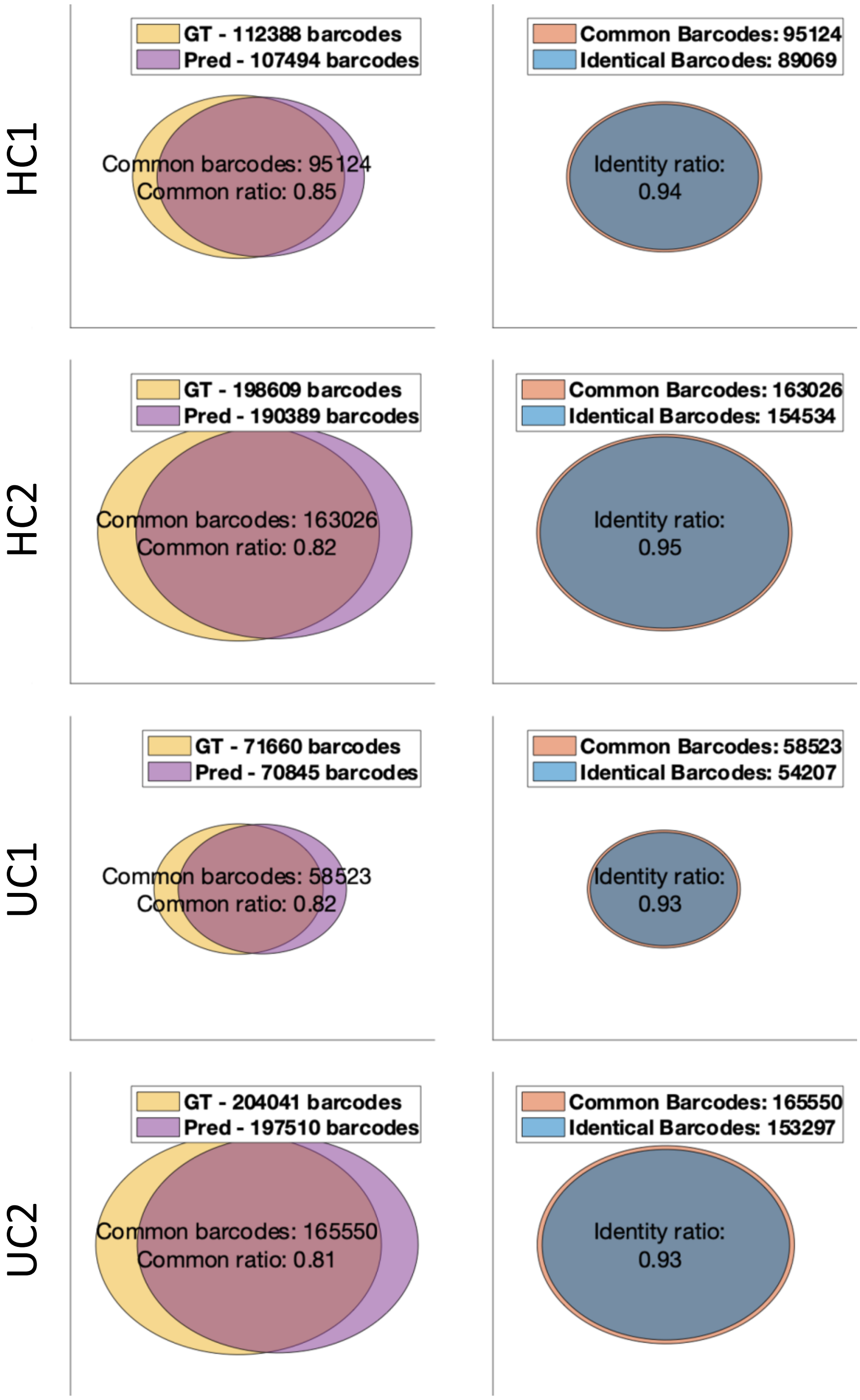

Figure S 9. Venn diagrams of barcode detection statistics in ground-truth (GT) versus Prediction (Pred). Left, Common barcodes are those that were eligibly read according to barcode criteria in the same position in both GT and Pred crops stacks (six detected markers, no adjacent identical markers, etc., as explained in the methods section), without comparing the actual barcode readout. Common ratio stands for common over the maximal eligible barcodes out of GT or Pred. Right, identical barcodes are the common barcodes that their color order exactly match between GT and Pred. Identity ratio stands for the identical over common barcodes.

Supplementary note 4: Error analysis of predicted barcodes readout

To follow the origin of DeepQR prediction errors, we analyzed the eligible barcode reads (i.e., reads that passed QC) that mismatched between the ground-truth and prediction. We compared the markers between ground-truth and prediction readouts for each mismatched barcode and registered all discrepancies. The distribution of read errors per marker color (figure S10) was calculated by considering only barcodes with a single marker mismatch to avoid over-representation of errors due to missed or falsely inserted markers (which create a permutation of the markers in the barcode read and therefore, excess error detections).

This analysis revealed that the main fraction of unsuccessful predictions was predominately attributed to an error in either green or yellow classification. We note that these errors may be mitigated by optimizing filter sets to register larger spectral windows (using laser-notch-filters such as NF03-405/488/561/635E by Semrock, instead of multi-band filters as used here) and a better bleed through correction.

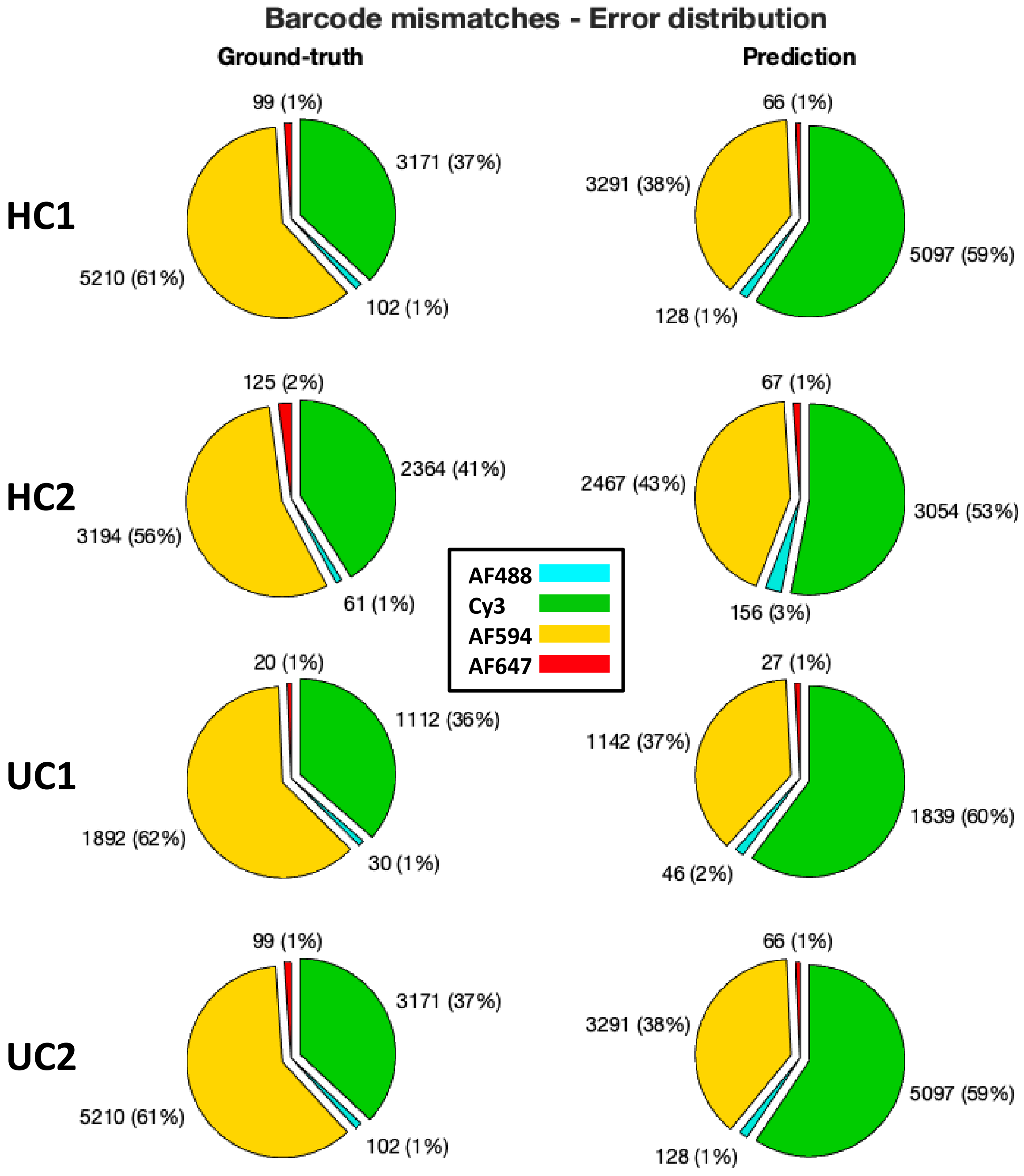

Mean (log2 expression)

Figure S 10. Barcode single mismatch error distributions. The distributions show all eligibly read barcodes (according to barcode criteria explained in the text, such as a total of six markers, no identical adjacent markers, etc.) in both ground-truth (GT) and prediction (Pred) that had a single marker mismatch between GT and Pred according to the marker's color. These distributions show that a major part of errors originate from Cy3-AF594 misclassifications, whereas misclassifications of AF488 or AF647 are extremely rare (<0.1% of a total number of eligible barcodes as presented in figures 2 and S9). Notably, in all samples, the prediction overestimates the abundance of Cy3 compared to AF594.

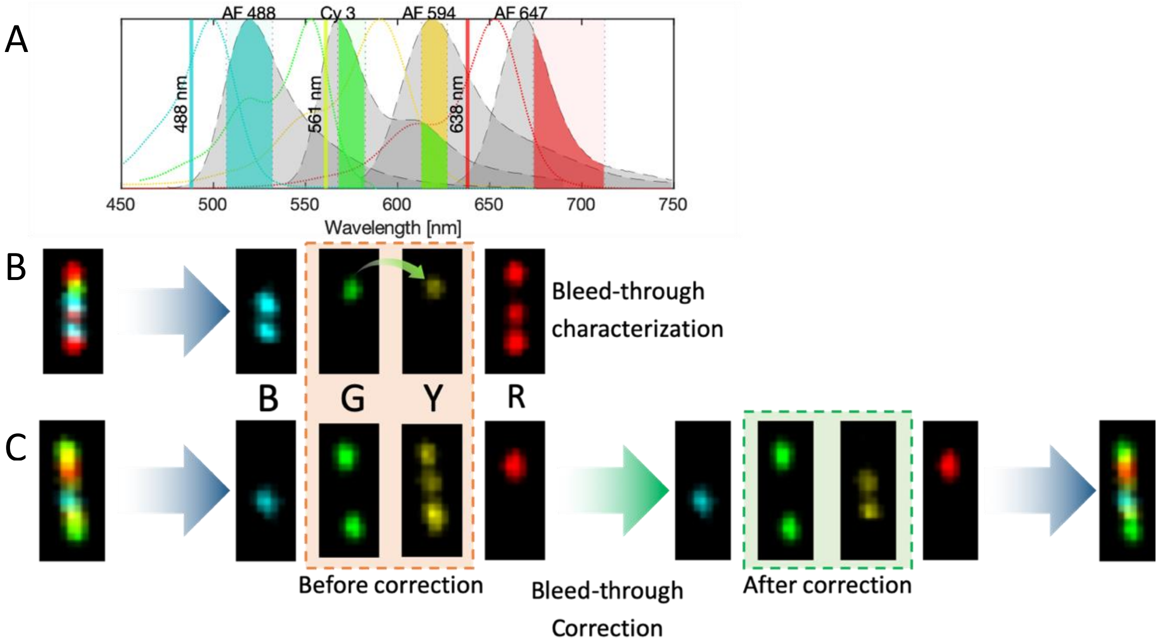

Figure S 11. Bleed-through correction pipeline (same for GT and prediction barcodes). A) Excitation (dotted colored lines) and emission (gray patches) spectra of the NanoString barcode dyes overlaid with the four different emission filters used to image the ground-truth channels (colored patches) and the excitation laser lines (solid vertical lines). As shown in the spectrum, due to the overlap of the 561nm laser excitation with both Cy3 (green- G) and AF594 (yellow-Y) excitation spectra, the Cy3 emission significantly bleeds-through to the AF594 channel (shown as overlay of the green patch in the AF594 emission window). B) An example barcode used for bleed-through characterization. As NanoString barcodes have strictly six color positions, barcodes presenting six distinct PSFs in the blue (B), green (G) and red (R) channels only were used to localize and estimate the average intensity, width and lateral displacement ratios between the green PSFs and their yellow bleed-through PSFs. C) The average parameters calculated over thousands of barcodes without any AF594 markers were later used to simulate effective bleed-through PSFs for each green PSF detection and to subtract it from the yellow channel, correcting the bleed-through. This correction allowed resolving adjacent green and yellow markers otherwise appearing as a single PSF (as seen before the correction at the bottom of the yellow channel in this example).

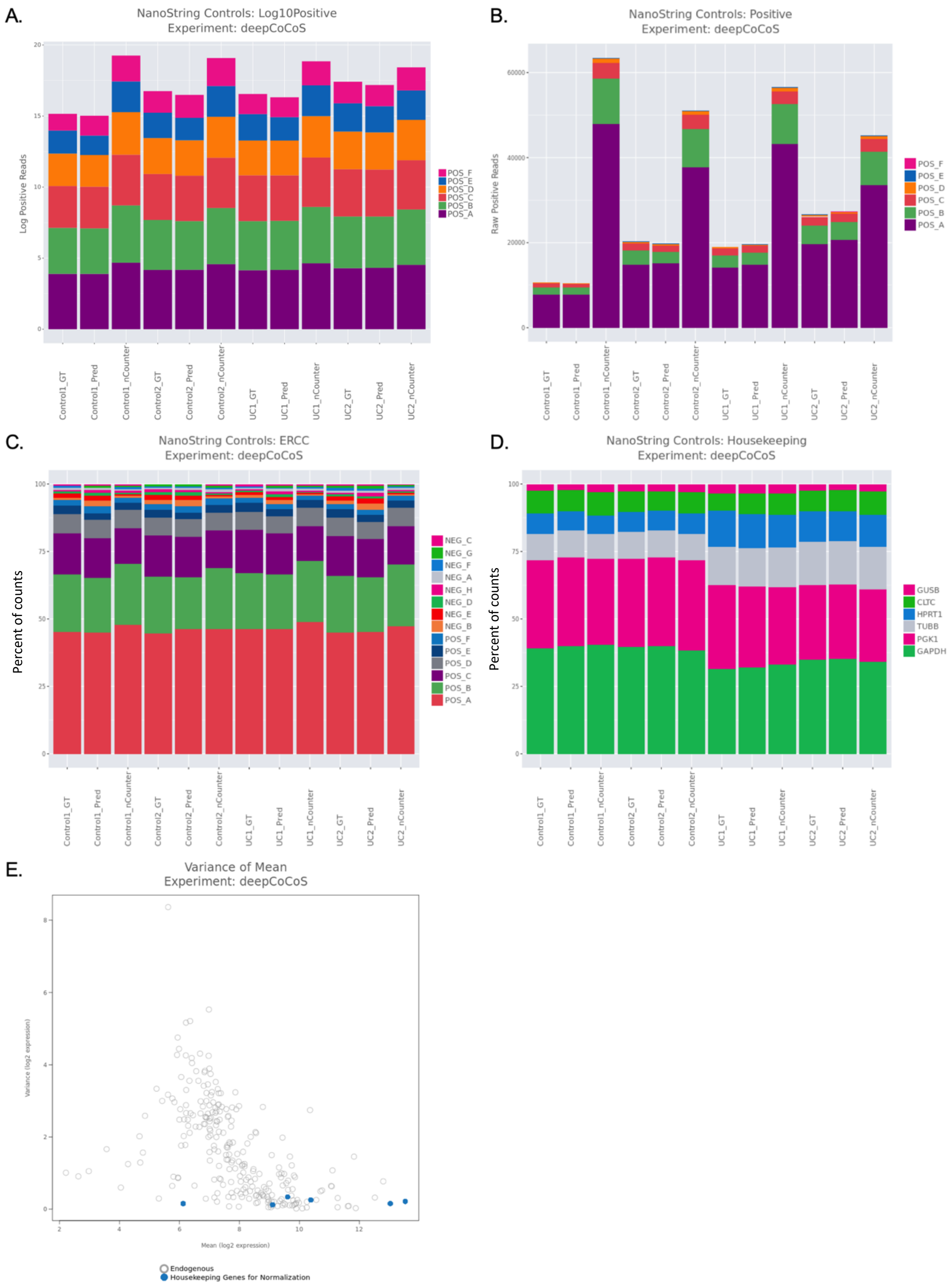

Figure S 12. Rosalind produced summary of the NanoString control barcodes readout across platforms and samples. A) log10 of positive control counts per sample and acquisition method showing the nCounter improved detection capability. B) As in A. displayed in absolute numbers (not log scaled). C) Percentage of spike-in control counts detection (both negative and positive controls). D) Percentage of housekeeping genes barcode detections, showing the similarity of housekeeping expression across methods and clinical states. E) Variance of counts across samples and platforms compared to the mean count value. This shows a low variance of the housekeeping genes (in blue) as required for their use in count normalization.

Supplementary note 5: **differential gene expression calculations**

Gene expression count normalization

In order to normalize the gene counts, the output gene counts tables from the barcode readout were converted to RCC files, which were further processed by the NanoString nSolver 4.0 software. We normalized all experimental results together: the nCounter, ground-truth and prediction RCC files in the same nSolver experiment. The normalization was performed according to the standard protocol with thresholding according to the geometric mean of negative controls, normalization according to the geometric mean of positive controls A-E (excluding POS_F due to a higher limit of detection in our custom detection analysis) and the standard CodeSet housekeeping genes normalization (according to the geometric mean of CLTC, GAPDH, GUSB, HPRT1, PGK1 and TUBB genes). The normalized results were exported as a text file for further analysis of the gene expression in Matlab.

Most differentially expressed genes

To calculate the most differentially expressed genes, the normalized expression results from the nSolver were processed with the “rnaseqde” Matlab function to give an adjusted p-value and log2 fold changes. The four samples were processed independently for each acquisition method (prediction, ground-truth and nCounter) and the three resulting gene expression tables were filtered for adjusted p-values smaller than 0.05. The tables were reordered according to gene fold-change scores, extracting the top 20 most differentially expressed genes in each one of the acquisition methods (see figures S13 and S14 and table S1). For comparison, the same analysis was performed using ROSALIND® barcode counts normalization and its differential expression analysis, resulting in slightly different results mainly due to different approaches for P-value calculation as described in the next section (see figures S16 and S17 and table S2). The Venn diagrams in figures S14 and S17 were produced in RStudio using the ‘venn.diagram’ function from the VennDiagram package.

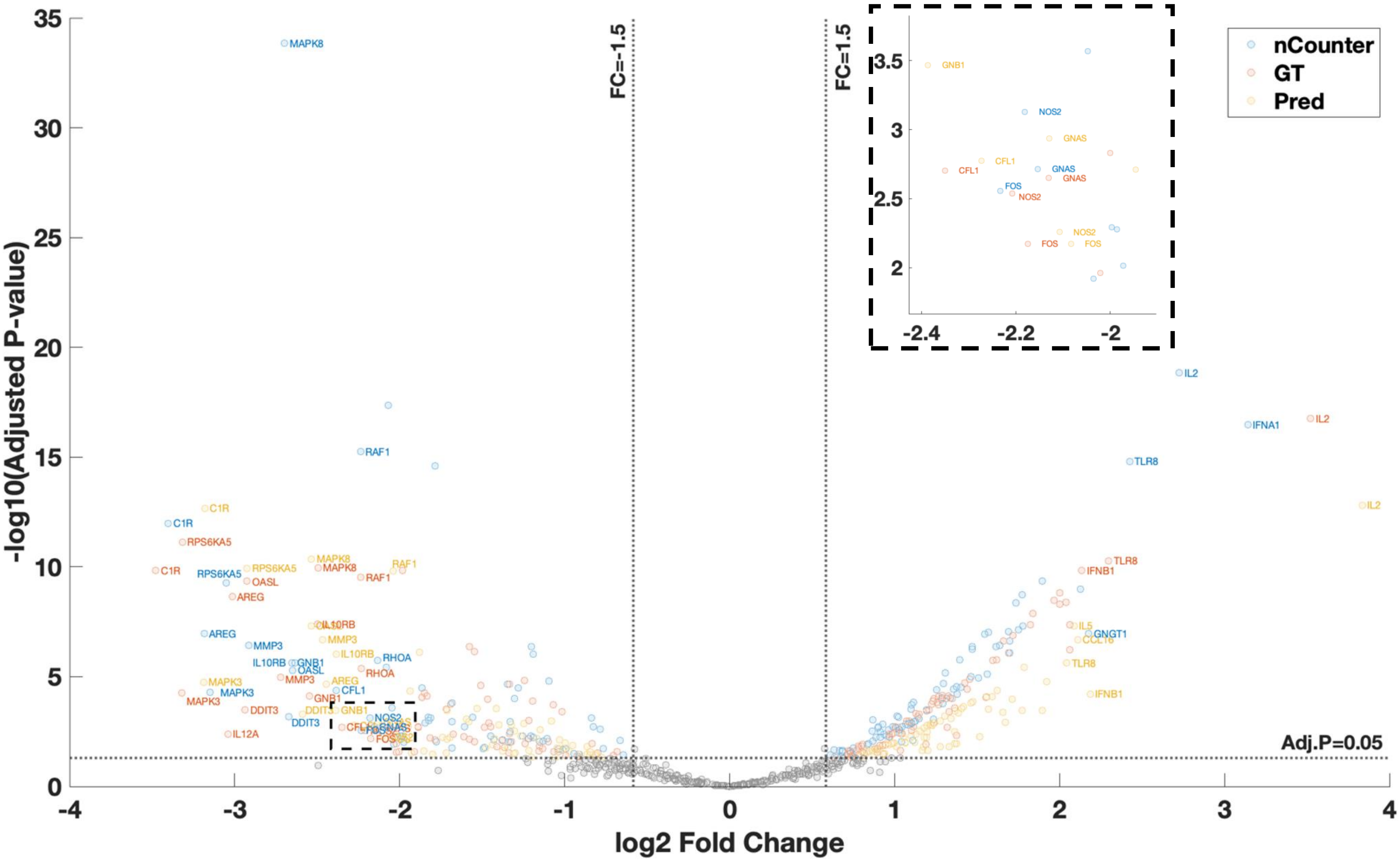

Figure S 13. Volcano plot of differential expression analysis using nSolver's normalization and Matlab's 'rnaseqde' function. The 20 most differential genes for each acquisition method (nCounter, GT and network prediction) are displayed by name. The inset shows a zoom-in of the portion of overlapping names marked with dashed rectangle.

nCounter						GT						Pred					
Probe Name	Log2 Fold Change	Mean Healthy Controls	Mean Patients with UC	P-Value	Adjusted P-Value	Probe Name	Log2 Fold Change	Mean Healthy Controls	Mean Patients with UC	P-Value	Adjusted P-Value	Probe Name	Log2 Fold Change	Mean Healthy Controls	Mean Patients with UC	P-Value	Adjusted P-Value
**C1R**	-3.40	1141.74	108.03	3.35E-14	1.04E-12	**IL2**	3.52	19.44	223.39	7.19E-20	1.79E-17	**IL2**	3.83	15.28	217.81	6.16E-16	1.53E-13
**AREG**	-3.18	2118.56	232.98	8.00E-09	1.10E-07	**C1R**	-3.48	1325.92	118.97	4.12E-12	1.47E-10	**MAPK3**	-3.19	3674.33	403.42	1.08E-06	1.80E-05
**MAPK3**	-3.15	3632.74	409.72	1.07E-05	5.31E-05	**MAPK3**	-3.32	4086.54	408.97	7.67E-06	5.46E-05	**C1R**	-3.18	1013.81	111.82	1.71E-15	2.13E-13
IFNA1	3.14	50.80	448.01	5.40E-19	3.36E-17	**RPS6KA5**	-3.32	2074.59	208.24	6.05E-14	7.54E-12	**RPS6KA5**	-2.92	1431.08	188.46	1.89E-12	1.18E-10
**RPS6KA5**	-3.05	1801.95	217.41	2.12E-11	5.27E-10	IL12A	-3.04	19.44	2.36	1.44E-03	4.06E-03	**DDIT3**	-2.59	5722.05	952.38	7.62E-05	5.13E-04
**MMP3**	-2.91	2649.44	351.32	3.54E-08	3.83E-07	**AREG**	-3.01	2087.32	258.29	1.03E-10	2.33E-09	**OASL**	-2.54	1529.94	263.80	1.28E-09	4.94E-08
**IL2**	2.73	39.06	258.34	1.18E-21	1.47E-19	**DDIT3**	-2.94	6264.39	818.90	7.80E-05	3.29E-04	**MAPK8**	-2.53	473.77	81.81	5.36E-13	4.45E-11
**MAPK8**	-2.70	638.66	98.45	5.79E-37	1.44E-34	**OASL**	-2.92	2019.17	266.05	1.55E-11	4.29E-10	**MMP3**	-2.47	1574.62	284.93	7.51E-09	2.08E-07
**DDIT3**	-2.67	5913.24	928.96	1.67E-04	6.61E-04	**MMP3**	-2.72	2371.88	359.81	1.17E-06	1.04E-05	**AREG**	-2.44	1865.78	343.02	1.46E-06	2.28E-05
**GNB1**	-2.65	2291.97	364.87	3.07E-07	2.32E-06	**GNB1**	-2.55	2874.80	491.97	1.13E-05	7.43E-05	**GNB1**	-2.39	2674.74	511.69	4.38E-05	3.40E-04
**OASL**	-2.65	2125.54	339.06	7.17E-07	5.10E-06	**IL10RB**	-2.50	1894.25	335.47	2.67E-09	4.15E-08	**IL10RB**	-2.38	1613.48	308.92	4.08E-08	9.23E-07
**IL10RB**	-2.63	2323.91	374.58	3.04E-07	2.32E-06	**MAPK8**	-2.49	559.95	99.36	1.75E-12	1.09E-10	**CFL1**	-2.27	11705.26	2422.15	3.53E-04	1.68E-03
**TLR8**	-2.50	16.86	2.99	3.80E-17	1.58E-15	**CFL1**	-2.35	12141.28	2381.25	6.36E-04	1.98E-03	IFNB1	2.19	25.76	117.26	4.78E-06	6.27E-05
**CFL1**	2.43	54.69	293.75	8.35E-06	4.24E-05	**TLR8**	2.30	54.94	270.11	6.35E-13	5.27E-11	**GNAS**	-2.13	3217.38	735.73	2.00E-04	1.16E-03
**RAF1**	-2.38	13181.60	2528.98	1.16E-17	5.79E-16	**RAF1**	-2.23	868.74	184.56	9.71E-12	3.02E-10	CCL16	2.11	64.91	280.04	6.82E-09	2.08E-07
**FOS**	-2.24	782.97	166.01	1.10E-03	2.79E-03	RHOA	-2.23	1490.80	317.37	4.50E-07	4.15E-06	**NOS2**	-2.11	13389.64	3107.43	1.47E-03	5.53E-03
**NOS2**	-2.23	8708.68	1852.84	2.05E-04	7.43E-04	**NOS2**	-2.21	14905.19	3226.09	9.58E-04	2.90E-03	IL5	2.09	84.14	357.77	1.39E-09	4.94E-08
GNGT1	-2.18	14102.44	3110.41	8.38E-09	1.10E-07	**FOS**	-2.17	11009.46	2438.31	2.61E-03	6.70E-03	**FOS**	-2.08	10222.32	2412.26	1.97E-03	6.71E-03
**GNAS**	2.18	29.00	131.03	6.95E-04	1.93E-03	IFNB1	2.13	69.20	303.93	3.77E-12	1.47E-10	**TLR8**	2.04	58.46	241.08	1.15E-07	2.39E-06
RHOA	-2.15	3267.95	734.62	2.21E-07	1.77E-06	**GNAS**	-2.13	3539.79	808.66	7.30E-04	2.24E-03	**RAF1**	-2.04	729.35	177.57	3.13E-12	1.56E-10

Table S 1. The 20 most differential genes according to Matlab's 'rnadeqde' function. Mean Patients and mean healthy columns correspond with an average of the normalized counts between the two corresponding samples, respectively. The presented data was calculated using Matlab's rnaseqde function as explained in the methods section of the main text. Bold gene entries in the table correspond with genes that are common to the 20 most differential gene lists in all three methods.

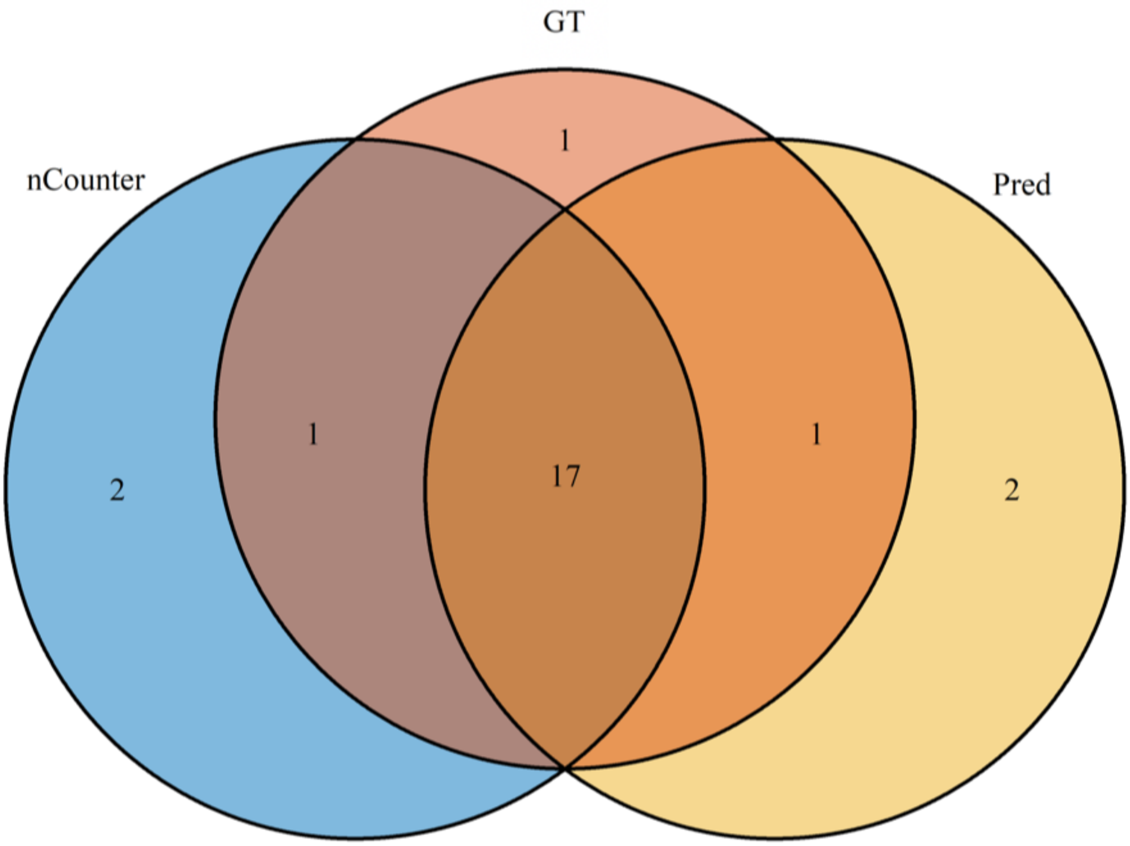

Figure S 14. Venn diagram of 20 most differentially expressed genes calculated with Matlab's 'rnaseqde' function across all detection methods.

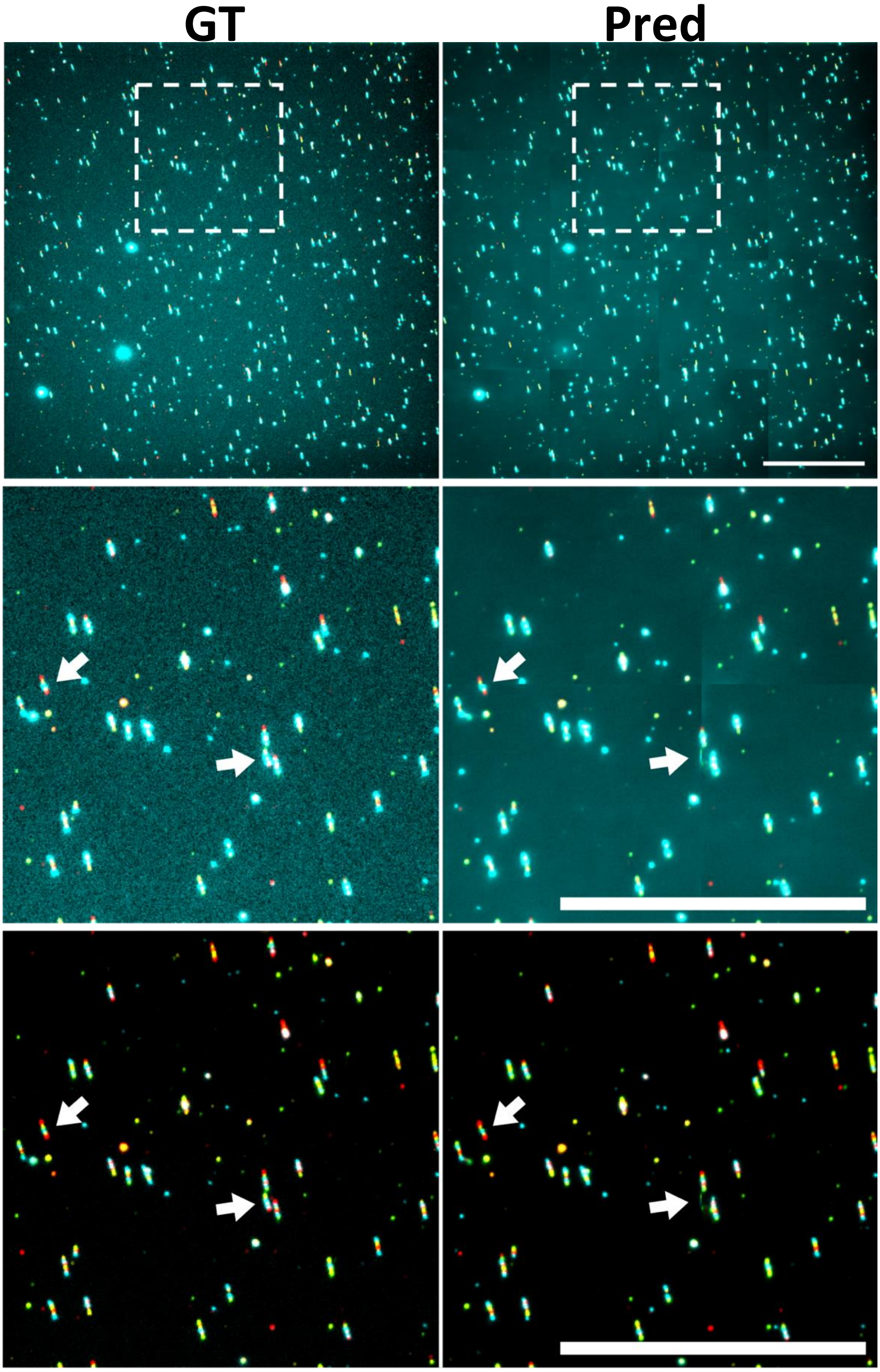

Figure S 15. Patch stitching artefacts. Top, an example full FOV with exaggerated brightness and contrast (B&C) levels to illustrate the patch stitching artefacts in the U-Net prediction result (right) compared to the same FOV in the GT (left), both images have the same B&C values in the blue channel. Middle, a zoom-in on the dashed rectangles regions in the above FOV, white arrows show examples for the effect these artefacts might have on barcode detection. Bottom, the same zoom-in as above but with adjusted B&C (same values between GT and prediction). All scale bars are 30 µm.

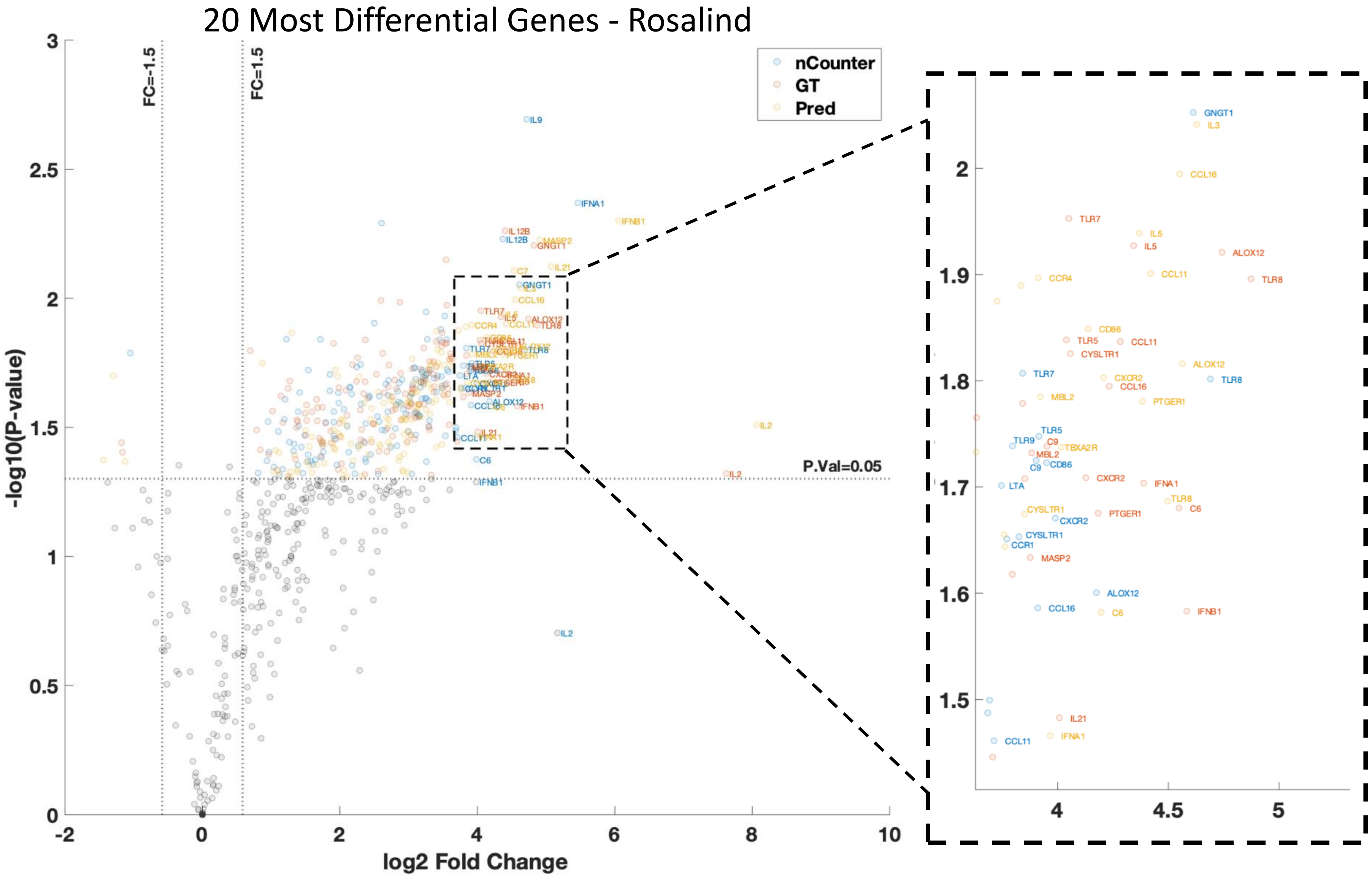

Figure S 16. Volcano plot of differential expression analysis using Rosalind's normalization and analysis. The 20 most differential genes for each acquisition method (nCounter, GT and network prediction) are displayed by name. The inset shows a zoom-in of the portion of overlapping names marked with dashed rectangle.

nCounter				GT				Pred			
Probe Name	Log2 Fold Change	P-Value	Adjusted P-Value	Probe Name	Log2 Fold Change	P-Value	Adjusted P-Value	Probe Name	Log2 Fold Change	P-Value	Adjusted P-Value
**IFNA1**	5.46	4.27E-03	7.26E-02	**IL2**	7.62	4.77E-02	7.57E-02	**IL2**	8.07	3.09E-02	8.17E-02
**IL2**	5.17	1.98E-01	2.18E-01	**TLR8**	4.87	1.27E-02	6.35E-02	**IFNB1**	6.06	5.00E-03	8.17E-02
IL9	4.72	2.03E-03	7.26E-02	GNGT1	4.82	6.24E-03	6.35E-02	IL21	5.08	7.54E-03	8.17E-02
**TLR8**	4.69	1.58E-02	7.26E-02	**ALOX12**	4.74	1.20E-02	6.35E-02	MASP2	4.91	5.94E-03	8.17E-02
GNGT1	4.61	8.86E-03	7.26E-02	**IFNB1**	4.58	2.61E-02	6.35E-02	IL3	4.63	9.09E-03	8.17E-02
IL12B	4.37	5.90E-03	7.26E-02	**C6**	4.55	2.09E-02	6.35E-02	**ALOX12**	4.56	1.53E-02	8.17E-02
**ALOX12**	4.17	2.51E-02	7.26E-02	IL12B	4.41	5.48E-03	6.35E-02	**CCL16**	4.55	1.01E-02	8.17E-02
**CXCR2**	3.99	2.13E-02	7.26E-02	**IFNA1**	4.39	1.98E-02	6.35E-02	C7	4.53	7.81E-03	8.17E-02
**C6**	3.99	4.21E-02	7.51E-02	IL5	4.34	1.18E-02	6.35E-02	**TLR8**	4.50	2.06E-02	8.17E-02
**IFNB1**	3.98	5.15E-02	8.11E-02	**CCL11**	4.28	1.46E-02	6.35E-02	**CCL11**	4.42	1.26E-02	8.17E-02
CD86	3.95	1.89E-02	7.26E-02	**CCL16**	4.23	1.60E-02	6.35E-02	PTGER1	4.38	1.66E-02	8.17E-02
TLR5	3.92	1.79E-02	7.26E-02	PTGER1	4.18	2.11E-02	6.35E-02	IL5	4.37	1.15E-02	8.17E-02
**CCL16**	3.91	2.59E-02	7.26E-02	**CXCR2**	4.13	1.96E-02	6.35E-02	**CXCR2**	4.21	1.57E-02	8.17E-02
C9	3.90	1.88E-02	7.26E-02	**CYSLTR1**	4.06	1.49E-02	6.35E-02	**C6**	4.20	2.62E-02	8.17E-02
TLR7	3.84	1.56E-02	7.26E-02	TLR7	4.05	1.11E-02	6.35E-02	CD86	4.14	1.42E-02	8.17E-02
**CYSLTR1**	3.83	2.22E-02	7.26E-02	TLR5	4.04	1.45E-02	6.35E-02	TBXA2R	4.02	1.83E-02	8.17E-02
TLR9	3.80	1.83E-02	7.26E-02	IL21	4.01	3.29E-02	6.35E-02	**IFNA1**	3.97	3.42E-02	8.17E-02
CCR1	3.77	2.23E-02	7.26E-02	C9	3.95	1.83E-02	6.35E-02	MBL2	3.92	1.64E-02	8.17E-02
LTA	3.75	1.99E-02	7.26E-02	MBL2	3.88	1.85E-02	6.35E-02	CCR4	3.91	1.27E-02	8.17E-02
**CCL11**	3.71	3.46E-02	7.31E-02	MASP2	3.88	2.33E-02	6.35E-02	**CYSLTR1**	3.85	2.12E-02	8.17E-02

Table S 2. The 20 most differential genes according to Rosalind's normalization and differential gene analysis. See https://www.rosalind.bio/en/knowledge/replicating-rosalind-analysis-in-nsolver-aa for full analysis details. Bold gene entries in the table correspond with genes that are common to the 20 most differential gene lists in all three methods.

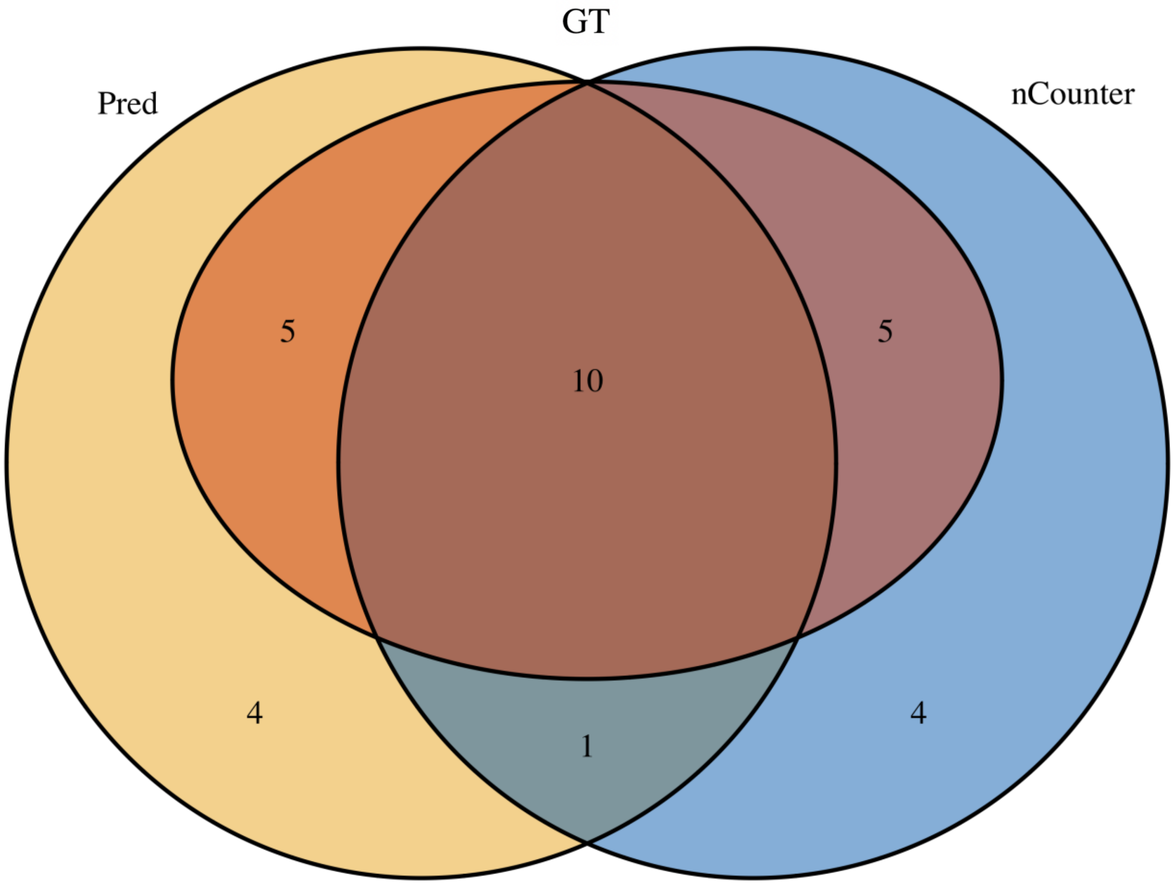

Figure S 17. Venn diagrams of 20 most differential genes according to Rosalind gene expression normalization and analysis.

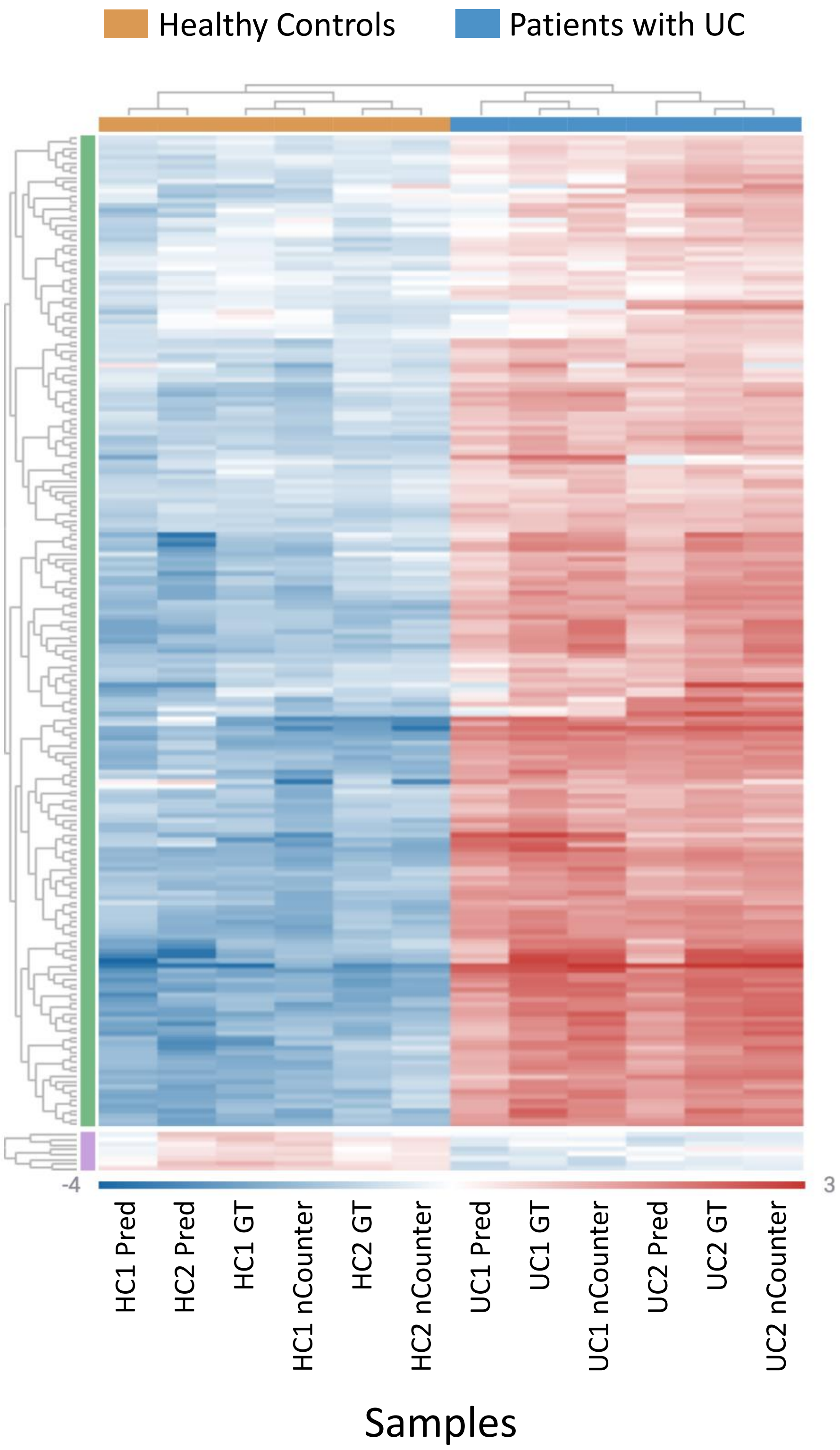

Figure S 18. Gene expression heatmap without using covariate correction of the detection method.

References:

1. Schindelin, J. *et al.* Fiji: An open-source platform for biological-image analysis. *Nat. Methods* **9**, 676–682 (2012).
2. Kingma, D. P. & Ba, J. L. Adam: A Method for Stochastic Optimization. *3rd Int. Conf. Learn. Represent. ICLR 2015 - Conf. Track Proc.* (2014). doi:10.48550/arxiv.1412.6980
